# Human Immunodeficiency Virus (HIV) Infection and Use of Illicit Substances Promote Secretion of Semen Exosomes that Enhance Monocyte Adhesion and Induce Actin Reorganization and Chemotactic Migration

**DOI:** 10.3390/cells8091027

**Published:** 2019-09-03

**Authors:** Yuan Lyu, Hussein Kaddour, Steven Kopcho, Tyler D. Panzner, Nadia Shouman, Eun-Young Kim, Jeremy Martinson, Heather McKay, Otoniel Martinez-Maza, Joseph B. Margolick, Jack T. Stapleton, Chioma M. Okeoma

**Affiliations:** 1 Department of Pharmacology, Stony Brook University School of Medicine, Stony Brook, NY 11794-8651, USA; 2 Division of Infectious Diseases, Department of Medicine, Northwestern University, Chicago, IL 60611, USA; 3 Department of Infectious Diseases and Microbiology, University of Pittsburgh Graduate School of Public Health, Pittsburgh, PA 15261, USA; 4 Department of Epidemiology, The Johns Hopkins Bloomberg School of Public Health, Baltimore, MD 21205, USA; 5 Departments of Obstetrics and Gynecology, and Microbiology, Immunology and Molecular Genetics, David Geffen School of Medicine at UCLA, Los Angeles, CA 90095, USA; 6 Department of Epidemiology, UCLA Fielding School of Public Health, Los Angeles, CA 90095, USA; 7 Department of Molecular Microbiology and Immunology, Johns Hopkins Bloomberg School of Public Health, Baltimore, MD 21207, USA; 8 Departments of Internal Medicine, Microbiology and Immunology, University of Iowa, Iowa City, IA 52242-1081, USA

**Keywords:** semen exosomes, monocytes, actin reorganization, psychostimulants, chemotaxis, morphometrics

## Abstract

Semen exosomes (SE) from HIV-uninfected (HIV−) individuals potently inhibit HIV infection in vitro. However, morphological changes in target cells in response to SE have not been characterized or have the effect of HIV infection or the use of illicit substances, specifically psychostimulants, on the function of SE been elucidated. The objective of this study was to evaluate the effect of HIV infection, psychostimulant use, and both together on SE-mediated regulation of monocyte function. SE were isolated from semen of HIV− and HIV-infected (HIV+) antiretroviral therapy (ART)-naive participants who reported either using or not using psychostimulants. The SE samples were thus designated as HIV−Drug−, HIV−Drug+, HIV+Drug−, and HIV+Drug+. U937 monocytes were treated with different SEs and analyzed for changes in transcriptome, morphometrics, actin reorganization, adhesion, and chemotaxis. HIV infection and/or use of psychostimulants had minimal effects on the physical characteristics of SE. However, different SEs had diverse effects on the messenger RNA signature of monocytes and rapidly induced monocyte adhesion and spreading. SE from HIV infected or psychostimulants users but not HIV−Drug− SE, stimulated actin reorganization, leading to the formation of filopodia-like structures and membrane ruffles containing F-actin and vinculin that in some cases were colocalized. All SE stimulated monocyte chemotaxis to HIV secretome and activated the secretion of matrix metalloproteinases, a phenotype exacerbated by HIV infection and psychostimulant use. SE-directed regulation of cellular morphometrics and chemotaxis depended on the donor clinical status because HIV infection and psychostimulant use altered SE function. Although our inclusion criteria specified the use of cocaine, humans are poly-drug and alcohol users and our study participants used psychostimulants, marijuana, opiates, and alcohol. Thus, it is possible that the effects observed in this study may be due to one of these other substances or due to an interaction between different substances.

## 1. Introduction

HIV encodes pathogenic proteins, such as gp120, Nef, and Tat, that modulate cellular architecture and behavior. Such modulations are implicated in HIV-induced pathological processes, including immune activation that persist during combination antiretroviral therapy (cART) and contribute to serious non-AIDS events. Although cART has dramatically reduced HIV/AIDS-related pathologies and mortality [1], use of illicit substances (mostly psychostimulants) is a major barrier to combating the HIV pandemic [2,3,4,5]. Psychostimulants such as cocaine have been linked to exacerbated HIV disease progression and HIV-associated disorders [6,7,8,9,10,11,12,13]. In addition to its ability to promote risky behavior [14], cocaine impairs antiviral mechanisms [8,15], thus increasing the risk of HIV acquisition. The combination of behavioral alteration and psychostimulant-mediated impairment of antiviral mechanisms continues to be a major obstacle in combating the global HIV/AIDS pandemic. The risk of exacerbated HIV disease progression and/or HIV-associated disorders among those who use psychostimulants and who are also infected with HIV is present in those adherent to cART [16,17,18].

Aside from the brain, peripheral tissues, including lymphocytes, monocytes, and the male urogenital organs, are responsive to psychostimulants due to the presence of dopamine transporters (DAT) and dopamine receptors (DR) [19,20,21]. In particular, DRD1 and DRD2 are expressed in male genital tissues such as the testis [22] and cocaine induces ultrastructural changes in the testis [23] and negatively affects testicular physiology as well as spermatogenic processes [23,24]. Similar to its function in the central nervous system, the function of dopamine in myeloid cells is mediated primarily by DRs, which are expressed in human monocytes and macrophages [25,26,27]. Myeloid cell DRs are functional and have been implicated in HIV infection and substance use disorders [28]. Although peripheral cells have been linked to increased viral replication in the presence of psychostimulants [29,30], how HIV and/or psychostimulants alter monocyte function is not completely understood.

Recently, acellular mechanisms regulating host functions have been discovered to occur through extracellular vesicles, in particular, exosomes, which are conveyors of bio-information [31,32,33]. Exosomes have been implicated in the modulation of immune responses [34,35] and microbial pathogenesis, including HIV infection [36,37,38,39,40,41,42,43,44,45]. Other biological processes, such as extracellular matrix (ECM) reorganization, epithelial barrier regulation, inflammatory cell recruitment, microglial migration [46], and regulation of HIV transcription [44], have been associated with exosomes [47]. Given that exosomes are released by various cell types into all body fluids [35,36,48,49,50,51,52,53], it is likely that HIV infection and/or psychostimulant-mediated effects on peripheral tissues may be imprinted in exosomes and that such exosomes may reprogram host gene expression and function. In a recent study, we showed that SEs from HIV-uninfected donors who do not use psychostimulants selectively modifies HIV-induced activation of host transcription factors [44].

In the present study, our goal was to evaluate the effect of HIV infection, psychostimulant use, and co-occurring HIV/psychostimulant use on SE-mediated regulation of monocyte function. We used monocytes as a model because monocytes are present in nearly every tissue, including the brain that has little or no T cell colonization. Moreover, monocytes differentiate into HIV target cells—dendritic cells and macrophages. Finally, monocytes are the first cells recruited to sites of inflammation, are one of the immune cell types present in semen [54,55], and are important target cells for mucosal HIV transmission [56], as well as HIV-associated neurocognitive disorders [57]. 

## 2. Materials and Methods

### 2.1. Ethics

This study involves the use of existing human specimens (semen) and, therefore, is not human subjects’ research. De-identified semen samples were obtained from participants in the Multicenter AIDS Cohort Study (MACS), a prospective cohort study of the natural history of HIV infection in men who have sex with men which was initiated in 1984 in 4 US sites and obtained semen samples from study participants semiannually from 1984 to 1987. The semen samples were stored at −80 °C until analysis in the present study. The participants included HIV− and HIV+ men who, at the time of collection, reported using or not using illicit substances. Studies were conducted according to University regulations approved by The University of Iowa and Stony Brook University Institutional Review Boards (IRB # 201608703). HIV-1-negative donors had no history of HIV, hepatitis B virus (HBV), or hepatitis C virus (HCV) infections. HIV-1-infected donors were ART-naive.

### 2.2. Semen Samples

A total of 64 samples from four clinical groups (HIV-uninfected and not illicit substance users, HIV−Drug−; HIV-uninfected and self-reported illicit substance users, HIV-Drug+; HIV-infected and not illicit substance users, HIV+Drug−; and HIV-infected and self-reported use of illicit substances, HIV+Drug+). A participant was classified as an illicit substance user only if they reported using cocaine (taken by any route); in other words, if a participant reported using other substances without cocaine, they were excluded (Table 1). Sixteen participants in each group were analyzed. The samples were received frozen on dry ice from the MACS. The samples were collected between 1984 and 1987, and participants were between 20 and 65 years old.

### 2.3. Cells

U937 monocytic cells were obtained from the American Type Culture Collection (ATCC) and maintained in complete Roswell Park Memorial Institute (RPMI) media (Corning, Thermofisher, Grand Island, NY, USA). HIV-1 LAV-infected HeLa CD4+ cells from which HIV secretome was collected were obtained from the National Institutes of Health (NIH) Aids Reagent Program and maintained in complete Dulbecco’s Modified Eagle Medium (DMEM) media. RPMI and DMEM media were supplemented with 10% fetal bovine serum (FBS) (Atlanta Biologicals, Flowery Branch, GA, USA) that was exosome-depleted by ultracentrifugation (100,000× *g*, 2 h, 4 °C), 1% Penicillin-streptomycin (Thermofisher, Grand Island, NY, USA), 1 µg/mL Amphotericin B (Thermofisher, Grand Island, NY, USA), 2 mM sodium pyruvate (Corning, Corning, NY, USA), 1% of glutamate (Thermofisher, Grand Island, NY, USA), and 10 mM 4-(2-hydroxyethyl)-1-piperazineethanesulfonic acid (HEPES) buffer (Fisher Biotech, Fair Lawn, NJ, USA) at pH 8. NucBlue™ Live ReadyProbes™ reagent was purchased from EasyProbes (Thermofisher, Grand Island, NY, USA). Cell Viability Imaging Kit (Blue/Green) was obtained from Genecopoeia (Rockville, MD, USA), and Type I collagen was purchased from Corning (Corning, NY, USA).

### 2.4. Isolation of Exosomes

64 semen samples from four clinical groups (n = 16/group) were liquefied at room temperature for 30 min and subsequently centrifuged at 10,000× *g* for 30 min to remove cellular debris and large vesicles. Clarified seminal plasmas were transferred to new tubes. For Nano Tracking Analysis (NTA) experiments, six pools of samples in each group, each pool from 2 participants (100 µL/sample), were used. Samples were pooled to obtain sufficient volume needed for efficient separation and analysis. For the rest of the experiments, 4 pooled samples (n = 16, 50 µL/sample) per clinical group were used. Exosomes were purified by size exclusion chromatography (SEC), where clarified seminal plasma was loaded onto Sephadex G-50 fine beads (GE-Healthcare, Pittsburgh, PA, USA) packed in a 22 cm × 1 cm Econo-column (Bio-Rad, Hercules, CA, USA). Elution was achieved by gravity using Phosphate Buffered Saline (PBS, Corning, NY, USA). Fractions of 200 µL were collected, and elution profiles were determined by absorbance measurements at 280 nm and 600 nm. The first peak which corresponds to semen exosomes (SE) was collected, and the protein content was measured by the Bradford Assay (Bio-Rad, Hercules, CA, USA). Of note, HIV could not be efficiently separated from semen exosomes using the Optiprep (Iodixanol)-based density gradient centrifugation method. While a good gradient prior to centrifugation was obtained, a satisfactory purification was not achieved due to the fact that the gold-standard exosomal marker AChE, as well as the exosomal markers CD9, CD63, and HSP70, along with the viral protein reverse transcriptase (RT) were found across the gradients. This is not surprising since HIV and exosomes overlap in size, density, and charge, and HIV is known to incorporate exosomal markers such as CD9, CD81 [58], and CD63 [59], while exosomes in turn also contain viral proteins [60] and RNA [61]. Immunocapture purification could not be used either because this mechanism depends on the use of antibodies against either host or viral proteins which are present in exosomes and HIV. Moreover, the “release” mechanism of exosomes trapped on the antibody-bead complex was inefficient. Thus, the inclusion of exosomal proteins in HIV and HIV proteins in exosomes hindered separation of these vesicles but also highlighted the need to assess the vesicles in their near-native state to understand their effect on host cells.

### 2.5. Nanoparticle Tracking Analysis (NTA)

Exosome size and concentration were measured by NTA using ZetaView PMX 110 (Particle Metrix, Mebane, NC, USA) and the corresponding software ZetaView v8.04.02. Samples were diluted appropriately in ultrapure water and measured under the same settings (temperature 25 °C, sensitivity 92, shutter speed 70, and frame rate 30 fps). Data acquisition for size and concentration was performed in triplicate measurements, and each replicate corresponded to 11 positions with two cycles of reading at each position. The system was aligned and calibrated with 102-nm polystyrene standard beads. After automated analysis of the 11 positions and removal of any outlier position, the median number (X50) was used to report the particle size. The measured concentration was normalized to the volume of plasma and reported in particles/mL of seminal plasma. For zeta potential, measurements were performed in ultrapure water (pH 5.8) and data were acquired in quintuplicate. Each replicate corresponded to two cycles of reading.

### 2.6. Transmission Electron Microscopy (TEM)

Microscopic analysis of exosome samples was performed as previously described [36,38]: 200 µL of purified SE were buffer exchanged with Tris buffer (pH = 7.5, 1 M) and concentrated through a 0.5-mL centrifugal filter (10,000 NMWL) into 50 µL; 10 µL of concentrated SE was applied on to carbon-coated copper grids (Pellco Easiglow, 0.2 mpar, 30 mA, 40 s, negative) and allowed to sit for 30 s. Excess samples were removed with filter paper. The grids were washed with distilled deionized water (ddH_2_O) twice, stained with 0.7% Uranyl Formate solution for 20 s, and then allowed to air dry. Images were viewed and collected using a FEI Tecnai12 BioTwinG 2 electron microscope. The samples were captured with an AMT XR-60 CCD Digital Camera system. The size of particles from TEM images were quantified by ImageJ.

Microscopic analysis of exosome samples was performed as previously described [36,38]: 200 µL of purified SE were buffer exchanged with Tris buffer (pH = 7.5, 1 M) and concentrated through a 0.5-mL centrifugal filter (10,000 NMWL) into 50 µL; 10 µL of concentrated SE was applied on to carbon-coated copper grids (Pellco Easiglow, 0.2 mpar, 30 mA, 40 s, negative) and allowed to sit for 30 s. Excess samples were removed with filter paper. The grids were washed with distilled deionized water (ddH_2_O) twice, stained with 0.7% Uranyl Formate solution for 20 s, and then allowed to air dry. Images were viewed and collected using a FEI Tecnai12 BioTwinG 2 electron microscope. The samples were captured with an AMT XR-60 CCD Digital Camera system. The size of particles from TEM images were quantified by ImageJ.

### 2.7. Reverse Transcriptase (RT) Assay

HIV RT activity was determined with an EnzCheck Reverse Transcriptase Assay kit (Invitrogen, Carlsbad, CA, USA) according to the manufacturer’s protocol. Briefly, 30 µg (~6 × 10^9^ particles) of purified SE (6 pools of 2 donors each for HIV+Drug− and HIV+Drug+ groups) were lysed with 6 µL Triton X-100 in a total volume of 50 µL per well, to which 20 µL of poly(A)-oligo(dT) in a polymerization buffer were added. Assay was performed in a 96-well black plate in triplicates. An equivalent volume of PBS was used as the negative control. RT standard curve was prepared with serial dilution (0, 0.625, 1.25, 2.5, and 5 µg/mL) of Murine Leukemia Virus (MLV) recombinant RT.

### 2.8. ELISA Assays

HIV p24 ELISA (Xpressbio, Frederick, MD, USA) and cocaine ELISA (Abnova, Taipei, Taiwan, China) were conducted by following the manufacturers’ protocols. Briefly, for HIV p24 ELISA, a total of 30 µg purified SE (~6 × 10^9^ particles) from HIV+ groups were tested in 6 pools of 2 donors each. An equivalent volume of PBS was used as the negative control. The same procedure was adopted for cocaine metabolite ELISA, with Drug+ groups being tested (6 pools of 2 donors each per group) in triplicate. The detection limit of the ELISA kit for HIV p24 and cocaine were 1.7 pg/mL and 1 ng/mL, respectively.

### 2.9. RNA Purification

Collagen coating of tissue culture plates was described previously [62]. Briefly, 6-well tissue culture plates were pre-coated with 50 µg/mL of collagen for 2 h at 37 °C, after which 1 mL of 2 mg/mL bovine serum albumin (BSA, Research Products International, Mount Prospect, IL, USA) was added to block nonspecific sites. Two million U937 cells treated with vehicle (PBS) or 100 µg/mL of SE from each of the four clinical groups were plated and incubated for 18 h at 37 °C and 5% CO_2_. Each treatment included three replicates. Subsequently, total RNA was extracted using the miRNeasy Mini Kit (Qiagen, Hilden, Germany) following the manufacturer’s protocol. An on-column DNAse digestion step (RNase-Free DNase set, Qiagen) was added after the first buffer wash step. The yield, quality, and size distribution of RNA isolated from the cells were determined using the Bioanalyzer instrument (Agilent, Santa Clara, CA, USA). Six hundred ng of the RNA from each treatment group was applied to an RNA Nano Chip, and the RNA profiles were detected and analyzed on the Agilent 2100 Bioanalyzer with 2100 Bioanalyzer expert software (v B.02.08.S1648 (SR 1)). The electropherogram traces and “gel-like” images were exported from the instrument’s software and presented in Supplementary Figure S1. Isolated RNA was used for microarray analysis or for cDNA synthesis and subsequent real-time quantitative PCR (RT-qPCR) analysis.

### 2.10. Microarray Analysis, Data Mining, and Data Visualization

150 ng of total RNA was prepared for microarray analysis using the GeneChip™ WT PLUS Reagent Kit (Applied Biosystems, Foster City, CA) according to manufacturer’s protocol. The samples were hybridized (16 h) to Clariom™ S Human Arrays (Applied Biosystems, Foster City, CA) in a GeneChip™ Hybridization Oven 645 (Applied Biosystems™). The arrays were washed and stained using the GeneChip™ Hybridization, Wash and Stain Kit (Applied Biosystems, Foster City, CA) in a GeneChip™ Fluidics Station 450 according to manufacturer’s protocol. The arrays were scanned in a GeneChip™ Scanner 3000 7G (Applied Biosystems, Foster City, CA). Quality control and initial analysis, including scatterplots of differentially expressed genes (DEGs), Venn analysis, and Kyoto Encyclopedia of Genes and Genomes (KEGG) pathway analysis, were performed using Transcriptome Analysis Console (TAC) v 4.0.0.25 (Applied Biosystems, Foster City, CA). Clustered heatmaps were plotted using heatmapper [63] (www.heatmapper.ca), with the average linkage method and Euclidean distance measurement method. The lists of SE, SE-Drug, and SE-HIV DEGs that were obtained from a TAC analysis were subjected to data mining in a Web-based Gene Set Analysis Toolkit (WebGestalt [64], www.webgestalt.org), from which biological process and molecular function and cellular component gene ontology (GO) terms were obtained.

### 2.11. Primer Design and Real-Time Quantitative PCR (RT-qPCR) Data Validation

Primers were designed using the Thermofisher oligoperfect program (https://www.thermofisher.com/us/en/home/life-science/oligonucleotides-primers-probes-genes/custom-dna-oligos/oligo-design-tools/oligoperfect.html). Primers were in silico validated using the University of California, Santa Cruz (UCSC) in silico-pcr program (http://mgc.ucsc.edu/cgi-bin/hgPcr). The designed primers are listed in Table 2. Five µg total RNA was used for cDNA synthesis using the High Capacity cDNA Reverse Transcription Kit (Applied Biosystems, Thermofisher). The cDNA was stored at −20 °C until use. The thermal cycler program and expression calculation was setup in a 7500 FAST real-time PCR system (Applied Biosystems, Thermofisher), and the fold change in gene expression was calculated using the standard ∆∆CT method.

### 2.12. Collagen Adhesion Assay

Flat-bottom 96-well plate were pre-coated with 50 µL of 50 µg/mL of Type I collagen (Corning, Corning, NY, USA) for 2 h at 37 °C; 40 µL of 2 mg/mL Bovine Serum Albumin (BSA) was used to block the nonspecific sites. Incoming U937 cells (10,000 cells/well) treated with 100 µg/mL of SE or with equivalent volume of vehicle (PBS control) were added to the pre-coated wells and allowed to adhere for 18 h at 37 °C. Non-adhered cells were gently washed off with PBS three times. The adhered cells were labeled with NucBlue™ for 20 min at room temperature, and the wells were imaged (4× objective) in their entirety in the DAPI channel (360 nm/460 nm excitation/emission) using a Lionheart FX Automated Microscope (BioTek, Winooski, VT, USA). The captured images were then stitched, and the cell numbers were calculated using Gen5 ImagePrime. Values were represented as the number of total adhered cells in each well. Each treatment included four repeated wells.

### 2.13. Evaluation of Cell Viability and Proliferation

A total of 10,000 U937 cells per well were seeded in a collagen-coated 96-well plate with 100 µg/mL of SE or equivalent volume of PBS for 18 h at 37 °C. All cells were collected after treatment and tested for viability by the Trypan Blue (Life Technologies, Carlsbad, CA, USA) exclusion and Live/Dead Cell Stain (Cell Viability Imaging Kit, GeneCopoeia, Rockville, MD, USA) methods. Cell proliferation was determined by counting the total number of live cells. The experiments were repeated 3 times, and each experiment included three replicates.

### 2.14. Immunofluorescence-Based Analysis of Cytoskeletal Changes and Focal Adhesion

U937 cells were plated (10,000 cells/well) on a 96-well glass bottom dish (Cellvis, Mountain View, CA, USA) coated with Type I collagen and treated with 100 µg/mL of respective SE. The plate was centrifuged at 200× *g* for 8 min to facilitate cellular adherence to the bottom of the well and incubated at 37 °C for 18 h. Following incubation, cells were washed with PBS and fixed with 4% paraformaldehyde (PFA) in PBS for 15 min. Cells were then permeabilized by incubation in 0.1% TritonX-100 for 10 min. AlexaFluor 594 Phalloidin (Thermofisher, Grand Island, NY, USA) and Alexa Fluor 488 Vinculin (Thermofisher, Grand Island, NY, USA) were applied in a 1:40 dilution for 1 h, followed by a 5-min DAPI stain. Images were acquired using a Lionheart FX Automated Microscope (Biotek, Winooski, VT, USA). Representative 10× and 60× images were acquired manually for five fields of view per well. Image procession was performed using Gen5 ImagePrime. Quantification of cellular size, area, and circularity was performed by Gen5 ImagePrime via masking of phalloidin. A circularity metric was created by inputting the equation $C=4\pi A/P^{2}$, where C is the circularity, A is the area of the cell, and P is the perimeter of the cell (https://imagej.nih.gov/ij/plugins/circularity.html).

### 2.15. Colocalization Analysis

60× fluorescent images of U937 cells treated with vehicle or SE (100 µg/mL) and stained with Alexa Fluor 594 Phalloidin and Alexa Fluor 488 Vinculin captured on a Lionheart FX Automated Microscope were imported into ImageJ (http://imagej.nih.gov/) for colocalization analysis. An open source ImageJ plugin “EzColocalization” (http://sites.imagej.net/EzColocalization/plugins/) was used to quantify colocalization of actin (phalloidin) and vinculin at regions of cell–cell contact and membrane protrusions. Regions of interests (ROIs) were selected via an ROI manager. Using EzColocalization, Pearson correlation coefficient (PCC) and Threshold overlap score (TOS, linear) quantifications were performed for 7 representative fields of view per SE treatment and vehicle. PCC and TOS (linear) values for each ROI were exported into GraphPad Prism for further analysis. One-way ANOVA was performed to determine the significance of SE treatment relative to the vehicle. Colocalization heatmaps were generated using the ImageJ plugin “Colocalization Colormap” (https://sites.google.com/site/colocalizationcolormap/home). 

### 2.16. Chemotaxis

Migration assays were conducted in a 10-well chemotaxis chamber (Neuroprobe Inc., Gaithersburg, MD, USA). Basal chambers were filled with media containing 0% FBS (serum-free), 30% FBS, or conditioned media from HIV-1 LAV-infected HeLa CD4+ cells (HIV secretome). A polycarbonate polyvinylpyrrolidone-free filter with a pore size of 5 µm was then placed over the lower chambers, and 285 µL of U937 cell suspensions (500,000 cells per well) that were pretreated for 24 h with either vehicle or 100 µg/mL SE from the 4 clinical groups in equal volumes of serum-free media were placed on the filter. The chambers were incubated for an additional 20 h at 37 °C in a 5% CO_2_ incubator. The apical chamber cells were carefully harvested, membranes were thoroughly rinsed, and basal chamber cells were harvested by piercing the membrane in the basal chamber. Cell suspensions were mixed with Trypan Blue dye, and total cell numbers and viability were quantified via hemocytometer counting.

### 2.17. Gelatin and Casein Zymography

After the 24 h serum starvation for migration assays, the conditioned media was harvested on ice. Following a 2000× *g* centrifuge step for 10 min, the media was mixed with a 4× Laemmli sample buffer (Bio-rad, Hercules, CA, USA) with the absence of boiling or 2-mercaptoethanol. Ten percent SDS-PAGE gels (0.75 mm thick) containing 0.1% gelatin in the resolving gel were prepared. Equal volumes of samples were loaded into the lanes, and electrophoresis was performed (Mini-PROTEAN Bio-Rad). Gels were removed from their cassettes, rinsed in distilled water, and incubated with a 1× Zymogram Renaturation Buffer (Bio-Rad) for 30 min with gentle agitation to remove SDS and to renature the proteins. Gels were then transferred to a 1× Zymogram Development Buffer (Bio-rad) for 30 min at room temperature, followed by replacement with fresh development buffer and incubated for 24 h at 37 °C to allow proteolytic digestion of the gelatin substrate. Gels were then rinsed with distilled water and stained with Coomassie blue for 30 min. Destaining was carried out with 50% methanol and 10% acetic for 1 h. Zones of gelatin degradation were imaged using an Odyssey CLx Imaging system (LI-COR Biosciences, Lincoln, NE, USA). The area of destained bands (zones of gelatin degradation) was then measured with ImageJ analyzing software and normalized to the value of vehicle treated samples. B-casein zymography was performed as described for gelatin zymography, aside from the inclusion of a 40 mA gel pre-running step performed prior to sample loading.

### 2.18. Statistical Analysis

The expression analysis settings for the microarray analysis were as follows: Gene-Level Fold Change < −2 or > 2, Gene-Level *p*-Value < 0.05, and ebayes ANOVA Method. The matrix correlation analysis was performed using GraphPad Prism software (v 8.1.2). Graphpad Prism was also used to plot all the graphs and to determine the statistical significance in this study. For a two-group comparison, unpaired t-test with Welch’s correction was used to determine the differences between the groups. For a four-group comparison, ordinary one-way ANOVA test with Dunnett’s correction was used in this study to determine the differences between SE groups as compared to HIV−Drug−. * *p* < 0.05, ** *p* < 0.01, *** *p* < 0.005, **** *p* < 0.001, and ns, nonsignificant.

### 2.19. Data Availability

The authors declare that all data supporting the findings of this study are available within the article. Microarray data have been deposited in the Gene Expression Omnibus (GEO) under accession code GSE129506.

## 3. Results

### 3.1. Biophysical Characterization of SE from Study Participants

**Figure 1:**
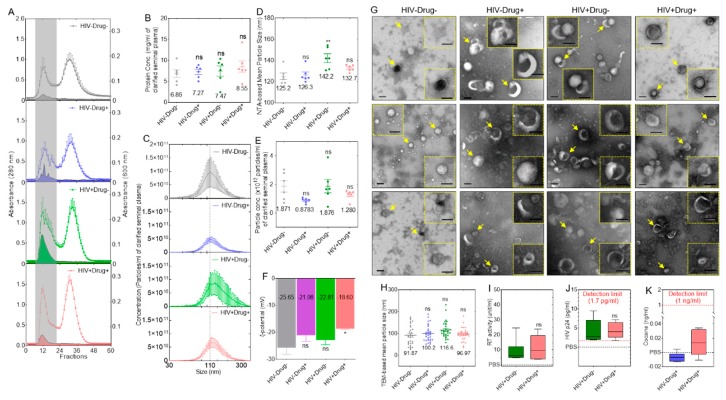
Physical characterization of semen exosomes (SE) from different clinical groups: (**A**) Exosomes were isolated from semen specimens obtained from donors in different clinical groups—HIV−Drug−, HIV−Drug+, HIV+Drug−, and HIV+Drug+. Seminal plasma from different participants were pooled, and the exosomes were purified by size exclusion chromatography (SEC). Six pools of clarified seminal plasma (n = 2, 100 µL/donor) from each of the four clinical groups were purified by size exclusion chromatography (SEC), and fractions were collected. UV–Vis was used to monitor absorbance at 280 nm and turbidity at 600 nm, indicative of the presence of proteins and lipid-containing vesicles, respectively. The dotted curve and filled curve represent absorbance profiles at 280 nm (protein) and 600 nm (lipid), respectively. The gray vertical rectangle highlights exosome fraction. (**B**) Purified SE fractions were pooled, and total protein concentration was determined by Bradford assay. Nano Tracking Analysis (NTA) measurements of the different SE physical properties: (**C**) size distribution profile, (**D**) NTA-based particle mean size, (**E**) mean particle concentration, and (**F**) Mean zeta potential (ζ-potential, mV). (**G**) Negative-stain TEM images of purified SE from the four clinical groups: The insets correspond to zoomed areas indicated by the arrow. All scale bars = 100 nm. (**H**) TEM-based mean particle size from Figure 1G determined with Image J. Assessment of HIV proteins: (**I**) HIV reverse transcriptase (RT) and (**J**) HIV p24. A total of 30 µg purified SE (~6 × 10^9^ particles) from HIV+ groups (6 pools of 2 donors each) were tested in triplicate. An equivalent volume of PBS was used as the negative control. (**K**) Cocaine metabolite ELISA. A total of 30 µg purified SE (~6 × 10^9^ particles) from Drug+ groups (6 pools of 2 donors each) were tested in triplicate. Equivalent volume of Phosphate Buffered Saline (PBS) was used as a negative control. The numbers in the graphs of Figure 1B,D–F,H indicate mean values. Error bars indicate SEM of 6 biological replicates. For a two-group comparison, unpaired t-test with Welch’s correction was used to determine the differences between the groups. For a four-group comparison, ordinary one-way ANOVA test (Dunnett’s correction) was used to determine the differences between the SE groups as compared to HIV−Drug−. * *p* < 0.05, ** *p* < 0.01, and ns, nonsignificant.

Ultraviolet–visible spectroscopy (UV–Vis) analysis of SE profiles indicated that the fractions eluted in the void peak were enriched in vesicles, whereas the vesicle-free proteins eluted in the latter peak (Figure 1A). Comparison of the profiles from the different clinical groups (n = 6) showed subtle differences in the height of the peaks (Figure 1A). The first peak, designated as SE, was collected and analyzed for protein concentration. No significant differences were observed in each of the SEs with protein concentrations ranging from 6.86 to 8.55 mg/mL of plasma (Figure 1B). NTA revealed subtle differences in SE size and concentration (Figure 1C) while the mean size of SE from HIV+Drug− was significantly different from the other groups (Figure 1D), the mean concentration was not different (Figure 1E). Since the electrical properties of the exosomal surface measured as ζ-potential is determined by the surface molecules on exosome membranes, we examined the effect of HIV infection and psychostimulant use on the ζ-potential of SE. Mean ζ-potential of HIV−Drug−, HIV−Drug+, and HIV+Drug− SE were not significantly different. In contrast, the ζ-potential of SE from HIV+Drug+ participants were significantly different from the HIV−Drug− SE (Figure 1F). TEM-based analysis showed no significant differences in SE morphology and size (Figure 1G,H).

### 3.2. HIV Proteins and Cocaine Metabolite (Benzoylecgonine) Are Associated with SE

RT assay and HIV p24 ELISA were used to assess the level of viral proteins associated with SE. The results showed mean values of 9.03 and 11.49 RT unit/mL for the HIV+Drug− and HIV+Drug+ groups, respectively (Figure 1I), whereas p24 mean values were 4.17 and 4.4 pg/mL for the HIV+Drug− and HIV+Drug+ groups, respectively (Figure 1J). Furthermore, the ELISA assay showed that detectable levels of cocaine metabolite benzoylecgonine is associated with SE, albeit below the assay detection limit of 1 ng/mL. Although the reasons for low levels of HIV proteins and benzoylecgonine in SE are unknown, it could be that the proteins and metabolites may be low in SE or that they are degraded, given the age of the seminal samples (~32 years) and the short half-life of benzoylecgonine, which is about 24 h for a single dose and a maximum of 10 days for chronic users [65]. It is also possible that SE do not carry this specific cocaine metabolite—benzoylecgonine. However, we were unable to assess other viral proteins and cocaine metabolites due to limited semen samples. Our data suggest that both HIV proteins and cocaine metabolite may be associated with SE. 

### 3.3. The effect of SE Stimulation on Gene Expression Signature of Collagen-Cultured Monocytes

**Figure 2:**
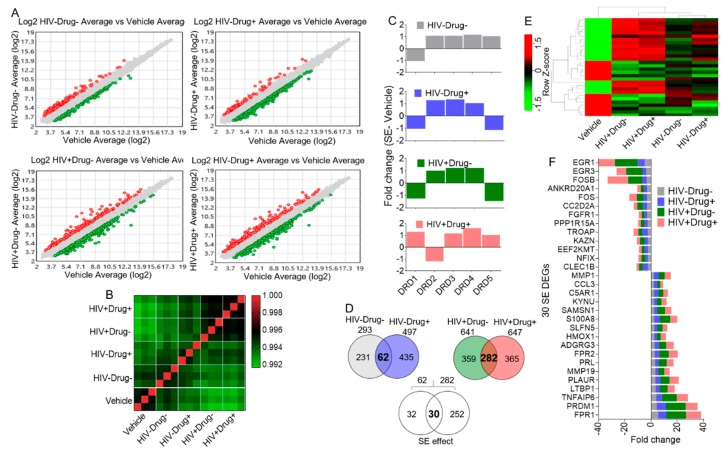
Microarray analysis of monocytes (n = 15) treated with vehicle or SE from different clinical groups (triplicate per treatment): (**A**) Scatter plots of the different SE treatments compared to the vehicle control. Red and green dots correspond to the significantly up- and downregulated genes, respectively. Gray dots correspond to the unchanged genes. (**B**) Correlation matrix of all genes (21448) showing a differential pattern in monocyte gene expression. (**C**) Gene expression levels of dopamine receptors as determined by microarray analysis. Differences were not significant with the set filtration criteria of fold change (FC) < −2 or FC > 2 and *p*-value < 0.05 (Benjamini and Hochberg correction for multiple observations). (**D**) Venn diagram analysis showing 30 differentially expressed genes (DEGs) common to all SE (HIV−Drug−, HIV−Drug+, HIV+Drug−, HIV+Drug+) treatments. The bold fonts in the Venn diagrams are the numbers of interest. (**E**) Hierarchical clustering heatmap showing the overall expression of the 30 SE-DEG. (**F**) Bar graph showing the fold change of each of the 30 SE-DEGs as compared to vehicle.

Since HIV infection and psychostimulant use did not significantly change the biophysical properties of SE, we examined the effect of SE from the different clinical backgrounds on the gene expression profile of monocytes cultured atop collagen as the substrate. We selected collagen as a relevant ECM because collagen is the most abundant matrix protein and HIV stimulates local production of collagen [66], which provides activating signals to myeloid cells, drives sustained inflammation, and alters the architecture of lymphoid tissues [67,68]. Figure 2A shows the distribution of all genes stimulated by the different SE in comparison to vehicle-treated cells. The correlation matrix, including all genes (21448) within the five treatment groups regardless of significance, shows that, in general, HIV−Drug− SE have minimal effect on the gene expression pattern of monocytes. However, psychostimulant use and/or HIV infection altered monocyte gene expression pattern (Figure 2B). Since monocytes express DRs which mediate cellular response to dopamine [69], we analyzed microarray data for levels of DR following treatment with the different SE. The results showed that monocytes express mRNA of DR variants (Figure 2C), although none of the SE significantly altered DR mRNA levels.

The effect of SE stimulation on monocytes gene expression profile was further delineated by identifying the differentially expressed genes (DEGs) shown in Table 3. Two-way Venn-filtration identified genes commonly altered by SE, regardless of the clinical group (Figure 2D). This analysis showed an overlap of 30 SE DEGs. Hierarchical clustered heatmap (Figure 2E) and a bar graph of fold change analysis (Figure 2F) was used to visualize the relationship between the genes, the direction of gene regulation, and the type of SE that elicited the regulation. In comparison to steady state levels of gene expression observed in vehicle-treated cells, 17 genes were upregulated by SE while 13 genes were suppressed by SE (Figure 2F). These results revealed specific gene expression signatures imprinted by SE from different clinical backgrounds. Among these DEGs are MMP1 and MMP19, which are proteins linked to the breakdown of extracellular matrix (ECM) in normal physiological (embryonic development, reproduction, and tissue remodeling) [70] and disease processes (arthritis, cancer metastasis, and HIV pathogenesis) [71,72]. In addition, the pro-inflammatory proteins CCL3, S100A8, and C5aR1 [73,74,75]; inflammatory regulators FPR1, FPR3, and TNFAIP6 [76,77]; cell proliferation, differentiation, and transformation regulatory proteins FOSB and FOS [78]; and transcription factors EGR1 and EGR3 [79] were also among the DEGs dysregulated by the different SEs. 

### 3.4. SE from Psychostimulant Users or HIV-Infected Participants Dysregulate Monocyte Gene Expression

**Figure 3:**
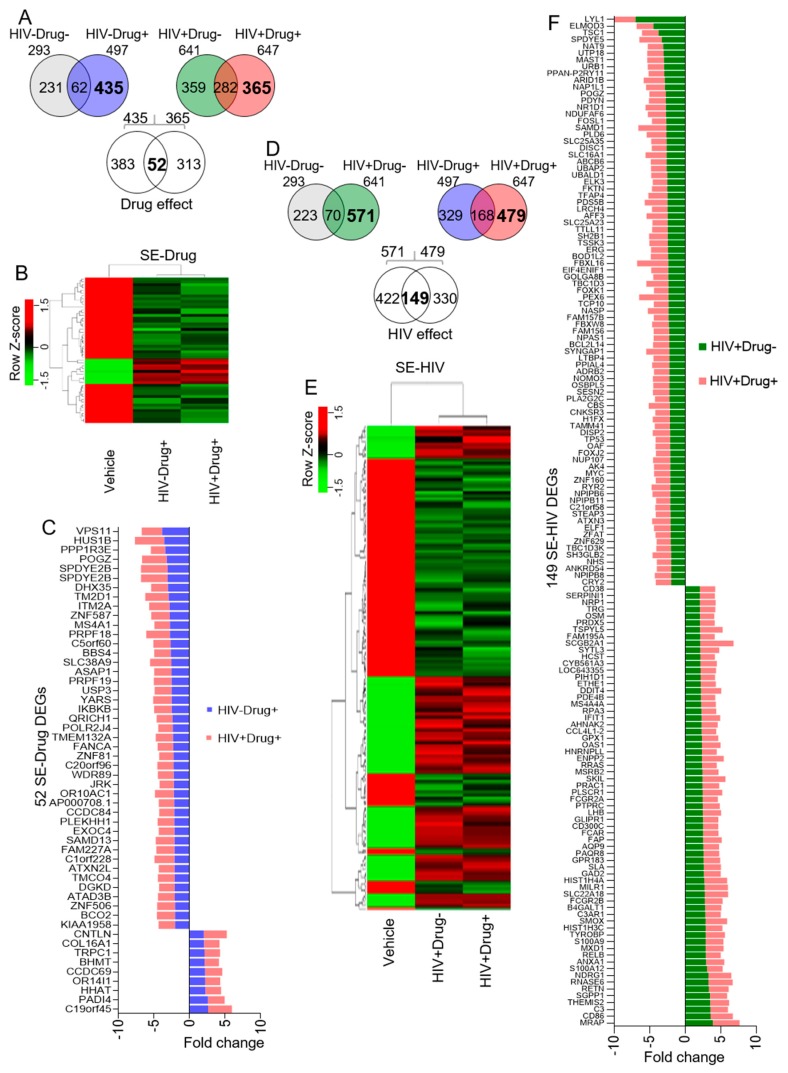
Microarray analysis of SE-treated monocytes showing the effect of psychostimulant use and HIV infection on SE function: (**A**) Venn diagram showing 52 DEGs exclusive to the alterations induced by treatment with HIV−Drug+ and HIV+Drug+ SE (SE-Drug). (**B**) Hierarchical clustering heatmap showing the overall and direction of expression of the 52 SE-Drug. (**C**) Bar graph showing the fold change of the 52 SE-DEGs as compared to vehicle. (**D**–**F**) Similar analyses as in Figure 3A–C showing 149 HIV exclusive DEGs in cells treated with HIV+Drug− and HIV+Drug+ SE (SE-HIV). The bold fonts in the Venn diagrams are the numbers of interest.

Two-way Venn-filtration was used to identify DEGs in cells treated with SE from HIV−Drug+ and HIV+Drug+ participants designated as SE-Drug DEGs (Figure 3A). Our analysis revealed that SE-Drug differentially regulated 52 genes, which is more than the 30 SE DEGs (Figure 2D–F). The relationship between the different genes induced by SE-Drug can be visualized using a hierarchical clustering heatmap (Figure 3B). Figure 3C shows individual genes within each of the SE-Drug DEGs and their expression patterns. A total of 9 genes were upregulated by SE-Drug while 43 genes were downregulated. Noteworthily, among the upregulated SE-Drug DEGs was collagen alpha-1(XVI) chain (COL16A1), known to be involved in inducing MMP9 secretion through AP-1 activation [80], in mediating cell attachment, in inducing integrin-mediated cellular reactions such as cell spreading and alterations in cell morphology [81,82], and in promoting glioma cell adhesion [83] and invasion [84]. Conversely, HUS1 Checkpoint Clamp Component B (HUS1B), which overexpression has been shown to induce cell death [85], was potently suppressed by Drug-SE. We used a similar approach to characterize gene expression changes induced by SE from HIV+Drug− and HIV+Drug+ participants (SE-HIV). The analysis identified 149 (65 upregulated and 84 downregulated) SE-HIV DEGs (Figure 3D), of which the hierarchical clustering (Figure 3E) revealed subtle differences between the two SE-HIV (HIV+Drug− and HIV+Drug+) groups compared to vehicle control. Fold differences and direction of gene expression between the SE-HIV groups are shown in Figure 3F. Among the 65 upregulated SE-HIV DEGs were inflammatory molecules, such as CCL4L1 and Resistin (RETN), transcription factors (RELB and MXD1), and antiviral molecules such as Interferon-induced antiviral RNA-binding protein (IFIT1) [86], whereas among the top downregulated SE-HIV DEGs was Tuberous Sclerosis 1 (TSC1), which is a tumor suppressor gene that maintains HIV-1 latency by negatively regulating the AKT-mTORC1 pathway [87].

### 3.5. Gene Ontology (GO) Analysis

**Figure 4:**
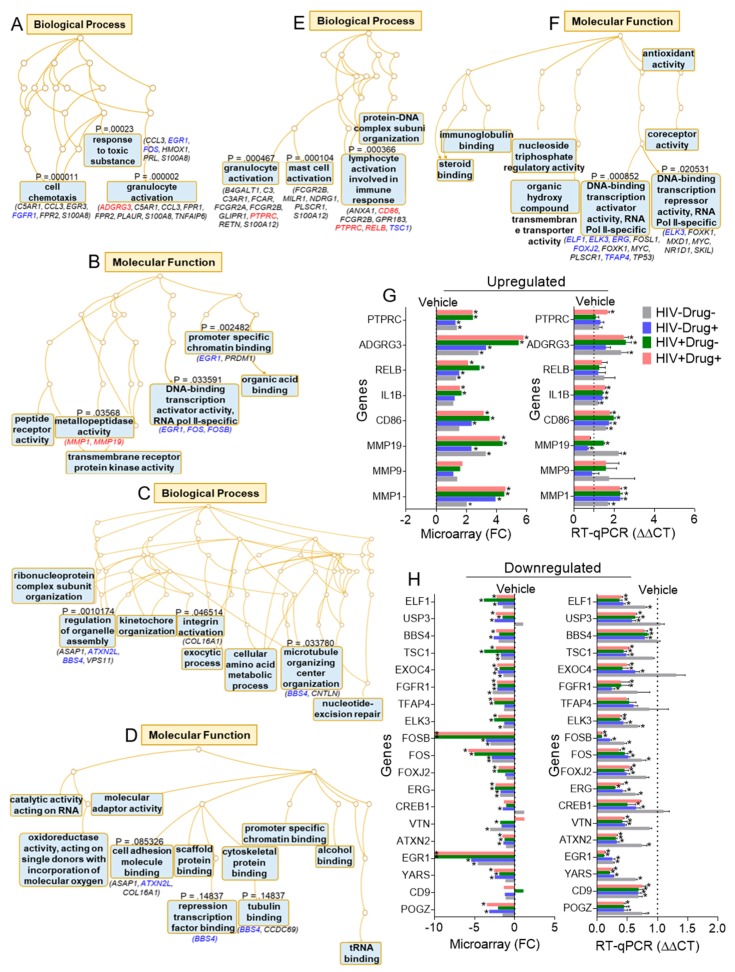
Gene Ontology (GO) terms and PCR validation of selected DEGs: Biological processes and molecular functions of selected DEGs as determined by the overrepresentation analysis method from the web-based GEne SeT AnaLysis Toolkit. (**A**,**B**) SE, (**C**,**D**) SE-Drug, and (**E**,**F**) SE-HIV. Colored fonts are validated genes with blue as downregulated and red as upregulated genes. (**G**,**H**) RT-qPCR validation of selected DEGs in Figure 4A–F (blue and red fonts). Ordinary one-way ANOVA test (Dunnett’s correction) was used to determine the differences between the SE groups as compared to HIV−Drug−. Error bars indicate standard deviation of three technical replicates. * *p* < 0.05, and nonsignificant labels were not shown for clarity.

We used GO enrichment analyses to predict the possible biological roles of the identified DEGs (Figure 4A–F). Table 4, Table 5 and Table 6 summarize the top 10 GO Terms enriched in three ontologies—biological processes (Figure 4A,C,E and Table 4), molecular function (Figure 4B,D,F and Table 5), and cellular component (Table 6). Furthermore, Table 7 listed the top 10 KEGG pathways identified by Webgestalt analysis. KEGG pathway by count, as determined by TAC software, identified focal adhesion as one of the top 10 common pathways in cells treated with SE from the four clinical groups (Table 8). 

### 3.6. Validation of Microarray Data

A subset of DEGs linked to two ontology terms—biological processes and molecular functions (Figure 4A–F, blue and red fonts)—were used for real-time quantitative PCR (RT-qPCR) validation using the primers shown in Table 2. The results show overall agreement between RT-qPCR and microarray results (Figure 4G), with subtle variation that may be attributed to assay differences. This validation study confirms that many genes were indeed differentially regulated by SE and that HIV infection, psychostimulant use, and HIV/psychostimulant use have the ability to regulate SE function.

### 3.7. SE from HIV-Infected Participants Who Used Psychostimulants Enhanced Monocyte Adhesion to Collagen

**Figure 5:**
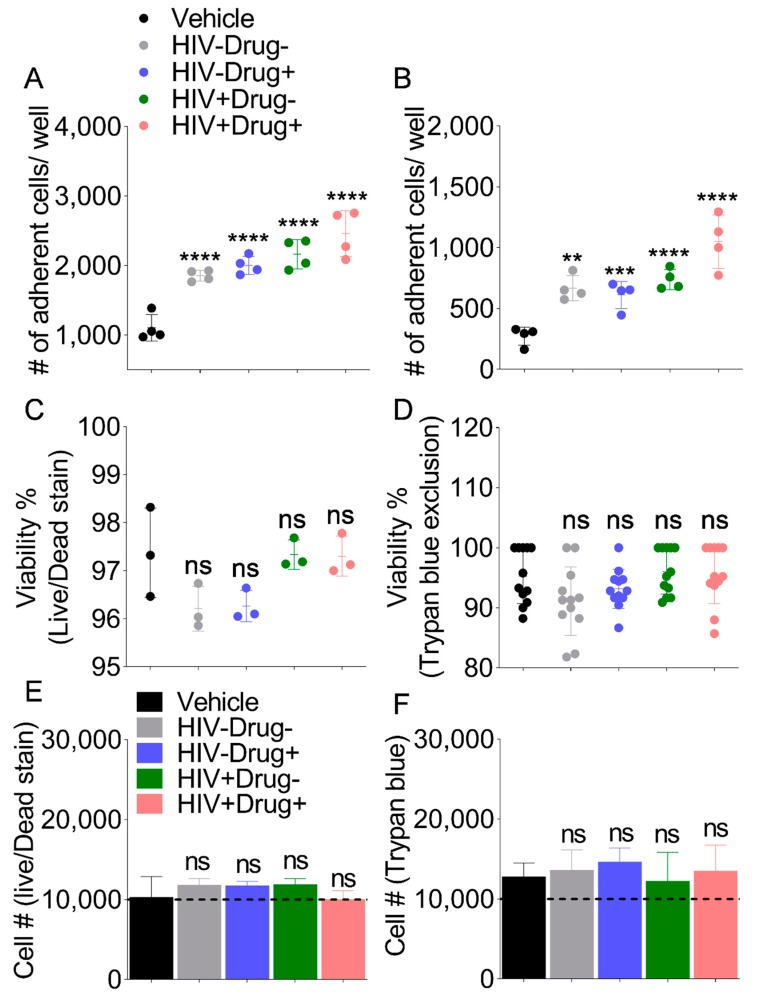
Monocyte adhesion to collagen is increased by SE from HIV-infected participants with comorbid psychostimulant use: 10,000 cells were treated with vehicle PBS or 100 µg/mL (~2 × 10^10^ particles/mL) SE from each clinical group and cultured atop collagen coated 96 well plate for 18 h in either complete media or serum free media. Subsequently, non-adhered cells were washed three times with PBS and adhered cells were stained with NucBlue. The entire well was imaged using LionHeart FX. The total number of adhered cells was determined using Gen5 Imaging software. Quantification of adherent monocytes in complete RPMI (**A**) and serum-free RPMI (**B**). Error bars indicate standard deviation of 4 independent wells per treatment. Analysis of monocyte viability by Live/Dead Stain (**C**) and Trypan Blue exclusion (**D**). Cell proliferation as determined by counting total live cells by Live/Dead Stain (**E**) or Trypan Blue stain (**F**). Error bars indicate standard deviation of three independent wells. Ordinary one-way ANOVA test (Dunnett’s correction) was used to determine the differences induced by the different SE treatments as compare to vehicle. * *p* < 0.05, ** *p* < 0.01, *** *p* < 0.005, **** *p* < 0.001, and ns, nonsignificant.

Since cell adhesion molecule binding is a molecular function GO term in gene expression analysis and COL16A1 involved in cell adhesion [81,82,83] is upregulated by SE-Drug (Figure 3C), we assessed the effect of SE on the adhesion of U937 monocytes to collagen. Monocyte adhesion occurred in untreated cells, with reduced adhesion when a serum-free medium was used. Compared to vehicle-treated cells, SE from all four clinical groups increased monocyte adhesion both in complete (Figure 5A) and serum-free (Figure 5B) media. However, adhesion of monocytes treated with HIV+Drug+ SE was the most enhanced. The increases in adhesion cannot be attributed to serum since similar increases were observed in serum-free conditions (compare Figure 5A,B). Furthermore, the increase in the number of adherent cells cannot be attributed to cell death or cell proliferation since none of the SE had cytotoxic (Figure 5C,D) or proliferative (Figure 5E,F) effects on U937 cells under the same experimental conditions. 

### 3.8. SE from HIV-Infected and Psychostimulant Users Induce Actin Reorganization

**Figure 6:**
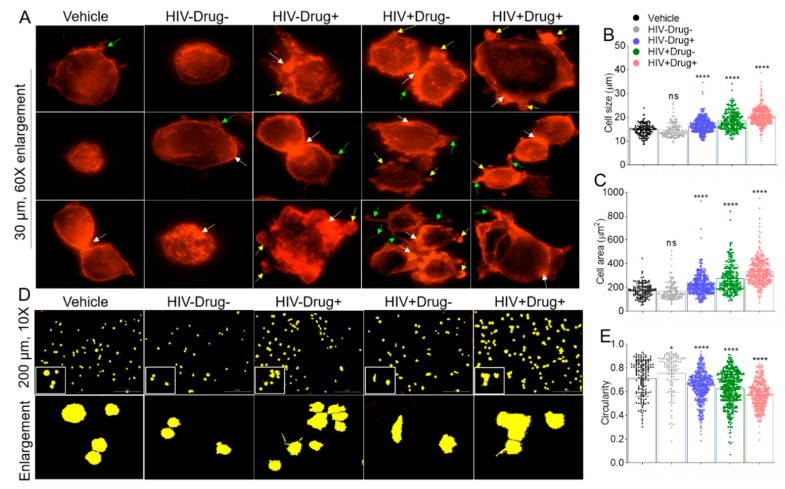
Altered monocyte morphometrics following treatment with SE from participants who used psychostimulants and/or were infected with HIV: (**A**) 60× fluorescence images of U937 monocytes plated atop collagen, treated with vehicle and different SEs as indicated in the figure, and stained with phalloidin to visualize actin membrane structures. Cells treated with SE-Drug or SE-HIV had distinct changes in morphology and exhibited significant membrane ruffles and filopodia-like extensions. White arrows indicate areas of increased F-actin localization. Green arrows indicate filopodia-like structures. Yellow arrows indicate membrane ruffles. Scale bar = 30 µm. The membrane tracing was used to assess monocyte circularity. The white box corresponds to the enlarged area. Scale bar is 200 µm. (**B**) Scatterplot of cell size. (**C**) Scatterplot of cell area. (**D**) Ten times representative membrane tracing (with fill) images of vehicle and different SE treatments obtained with the Lionheart FX Gen5 software. (**E**) Scatter plot of cell circularity. Calculations were performed by Lionheart FX Gen5 software. Ordinary one-way ANOVA (Dunnett’s correction) was used to determine the significance of SE treatments relative to vehicle. * *p* < 0.05, ** *p* < 0.01, *** *p* < 0.005, **** *p* < 0.001, and ns, nonsignificant.

To further understand the effects of SE on monocyte adhesion to collagen, we examined cell morphology and cytoskeletal dynamics of SE-treated cells compared to vehicle-treatment. SE from different clinical groups induced distinct changes in cell morphology and actin organization in monocytes (Figure 6A). When unstimulated or HIV−Drug− SE-treated monocytes were allowed to adhere to collagen-coated coverslips, the majority of cells maintained a cortical ring of actin filaments (Figure 6A, columns 1 and 2). In the presence of SE-Drug and SE-HIV, monocytes displayed considerable degrees of spreading and polarization (Figure 6A, columns 3 to 5) with actin localized to membrane ruffles and areas of cell-to-cell contacts (Figure 6A, columns 3 to 5). SE-polarized cells displayed asymmetric morphology suggestive of cell migration, with cell periphery displaying membrane ruffles and filopodia-like structures (Figure 6A). We measured the dynamics of SE on cell spreading by quantifying the cell size and cell area. Cells treated with vehicle had an average size of 14.74 µm, with a spread cell area of 172.48 μm^2^ (Figure 6B,C). The size (14.58 μm) and area (170.63 μm^2^) of cells treated with HIV−Drug− SE were similar to vehicle-treated cells. At variance, HIV−Drug+, HIV+Drug−, and HIV+Drug+ SE significantly increased cell sizes to 16.65, 18.49, and 20.5 µm respectively, and cell areas to 221.33, 274.16, and 333.70 μm^2^ respectively (Figure 6B,C). To further assess monocyte morphometrics, we traced the cell membrane (Figure 6D) and determined monocyte circularity. Monocytes are circular cells, and the circularity of cells treated with vehicle was highest at 0.71, with 1 being a perfect circle. In contrast, average cell circularity increased to 0.76 in cells treated with HIV−Drug− SE. However, treatment of monocytes with HIV−Drug+, HIV+Drug−, or HIV+Drug+ SE decreased monocyte circularity to 0.65, 0.63, and 0.58 respectively (Figure 6E). These data further support the induction of membrane ruffles and filopodia-like structures in monocytes treated with SE-Drug or SE-HIV and suggest that infection with HIV or use of psychostimulants alter the function of SE. Noteworthy is that filopodia and other thin membrane protrusions are sensitive to PFA fixation [88]. Since our cells were fixed, we may have underestimated the extent of membrane protrusions on the cells. Thus, studies of the effect of SE on monocyte morphometrics using unfixed living cells is warranted and not conducted in the present study due to limited semen specimens from all clinical groups. However, our data suggest that SE-Drug and SE-HIV induce monocytes to polarize.

### 3.9. SE from HIV Infected and Psychostimulant Users Enhance Monocyte Chemotaxis

**Figure 7:**
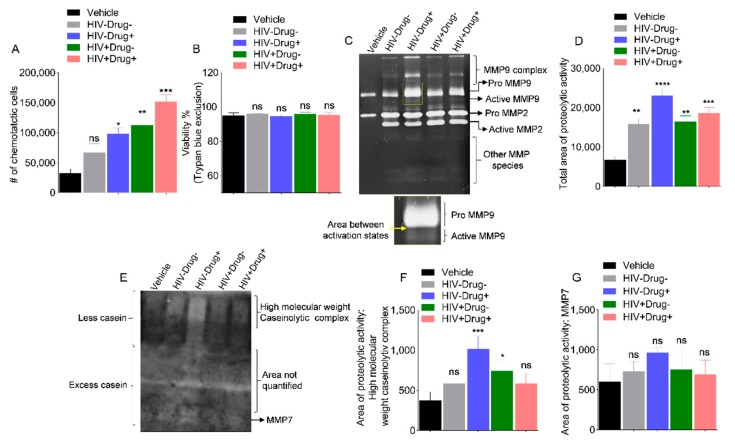
Monocyte chemotaxis and secretion of matrix-modifying enzymes are potentiated by SE from HIV-infected and HIV-uninfected participants with co-occurring psychostimulant use: 500,000 U937 cells were treated with vehicle PBS or 100 µg/mL SE from the 4 clinical groups in serum-free media for 24 h before addition to the apical side of a chemotaxis chamber, containing 0% FBS (serum-free), 30% exosome-depleted FBS, or conditioned media from HIV-1 LAV-infected HeLa CD4+ cells (HIV secretome). The chambers were incubated for 20 h at 37 °C in a 5% CO_2_ incubator. The basal chamber cells were harvested and quantified by Trypan Blue hemocytometer counting. (**A**) Monocytes treated with the different SE were assessed for chemotaxis toward clarified supernatants collected from HIV-infected cells (HIV secretome), and the numbers of monocytes that migrated into HIV secretome in the basal compartment of the migration chamber are shown. (**B**) Viability of unmigrated cells in the apical chamber at the end of the experiment expressed as % of viable cells. (**C**) Representative (n = 3) gelatin Zymograph of conditioned media (secretome) from monocytes treated with the various SE prior to loading into migration chambers. The area within the square box is enlarged below to highlight active MMP9. (**D**) Image J quantification (total densitometry) of total gelatinolytic activity of each zymogram lane presented as the total area of proteolytic activity. Data represent the mean of triplicate experiments, and error bars are standard error of the mean. (**E**) Representative (n = 3) β-casein zymography of the same conditioned media used in gelatin zymography. Image J quantification (densitometry measurement) of Figure 7F’s (**F**) high molecular weight caseinolytic complex and (**G**) MMP7 activities of each zymogram lane presented as area of proteolytic activity. Experiments were repeated three times with similar trends. Ordinary one-way ANOVA (Dunnett’s correction) test was used to determine the significance of different SE treatments relative to vehicle. * *p* < 0.05, ** *p* < 0.01, *** *p* < 0.005, **** *p* < 0.001, and ns, nonsignificant.

Given the reported enhanced adhesion of monocytes to endothelial surfaces in the context of HIV infection [89,90] and our observation that SE-Drug and SE-HIV modified monocyte cytoskeleton and exacerbated their adhesion to collagen, we sought to gain further insights into the effect of SE from psychostimulant users and HIV-infected subjects on monocyte motility. In general, while all SE induced chemotactic migration, HIV+Drug+ had the highest induction. In comparison to vehicle, HIV−Drug− SE modestly induced monocyte chemotaxis to HIV secretome, but chemotaxis was significantly higher in cells treated with SE from HIV+Drug− (20-fold increase), HIV−Drug+ (~19-fold increase), and HIV+Drug+ (~33-fold increase), in that order (Figure 7A). The differences in chemotaxis is independent of cell viability (Figure 7B), suggesting that HIV infection and psychostimulants use may reprogram SE to induce monocyte motility. 

### 3.10. HIV Infection and Psychostimulant Use Enhanced Secretion of SE that Activated Matrix Metalloproteinases

Various gelatinolytic degradative areas corresponding to active and pro MMP2 and MMP9, along with other MMP species were observed in secretomes of SE-treated cells compared to vehicle-treated cells (Figure 7C). The highest gelatinolytic activity measured by the total degradative area was 3.4-fold compared to vehicle-treated cells, and this activity was observed in th secretomes of cells treated with HIV−Drug+ SE (Figure 7D). Similarly, secretomes from SE-treated cells contain caseinolytic enzymes (Figure 7E). For both gelatin and casein zymographs, the cleared bands indicate areas of enzymatic activities while the arrows/brackets indicate various MMP species based on the correspondence of their size (kDa) to known MMP sizes. Casein zymography is technically challenging and less sensitive than gelatin zymography (compare Figure 7C,E). This is partly because of the migration pattern of casein which results in two clearly defined zones in the gel (Figure 7E, less and excess casein), the lower part that contains excess casein and the upper part with less casein. This is problematic because some MMPs, such as the pro (~28 kDa) and active (~20 kDa) forms of MMP7, migrate near the casein-migration boundary and were difficult to distinguish. While gelatin zymography predominantly detects MMP2 and MMP9 activities, casein is a preferential substrate for MMP3, MMP7, MMP12, and MMP13 [91,92,93]. Quantitation of select caseinolytic degradative areas showed that HIV−Drug+ SE-treated cells produced increased amounts of high molecular weight caseinolytic complexes (Figure 7F) with subtle differences in MMP7 (Figure 7G). Collectively, these results provide evidence of SE-induced migration and ECM-modifying MMPs that degrade gelatin (denatured form of collagen) and β-casein. However, whether SE-mediated cell migration and activation of MMPs play a role in immune surveillance, viral pathogenesis, maintenance, and/or disruption of barrier integrity remains to be determined. It also remains to be determined what role, if any, SEs play in activating lipases and other proteases.

**Figure 8:**
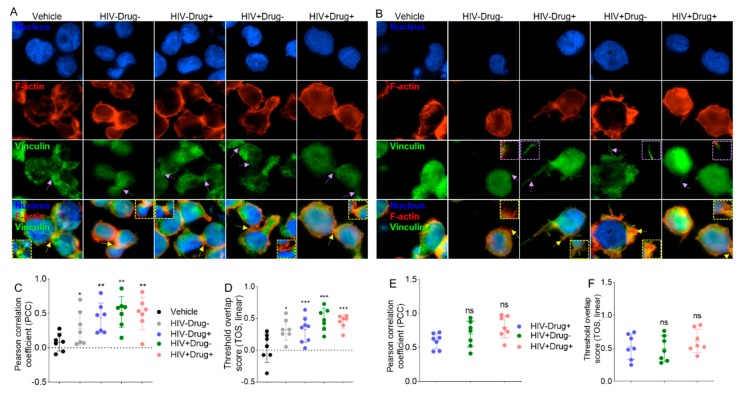
SE induced F-actin•Vinculin colocalization in monocytes. Representative 60× fluorescence images of U937 cells stained with phalloidin and vinculin to visualize (**A**) F-actin•Vinculin colocalization in adherence junctions. (**B**) F-actin•Vinculin colocalization in cell protrusions. Scale bar = 30 µm. Pearson correlation coefficient (PPC) and threshold overlap score (TOS, linear) plots corresponding to the images in Figure 8A,B for comparison of F-actin•Vinculin colocalization in adherence junctions (**C**,**D**) and cell protrusions (**E**,**F**). Purple arrows indicate vinculin in AJs (Figure 8A) or in areas of cell protrusion (Figure 8B). Yellow arrows indicate F-actin•Vinculin complex in AJs (Figure 8A) and areas of cell protrusions (Figure 8B). Ordinary one-way ANOVA (Dunnett’s correction) was used to assess statistical significance relative to vehicle. * *p* < 0.05, ** *p* < 0.01, *** *p* < 0.005, and ns, nonsignificant.

HIV infection and psychostimulant use promote secretion of SE that enhanced formation of focal adhesions and adherence junctions: Since formation of focal adhesion (FA) is integral to cell adhesion and migration, we examined the effects of SE on FA using the general adhesion proteins vinculin and F-actin, which form part of the cell’s cytoskeleton. While vinculin localized to cell-to-cell contacts (adherence junctions, AJ) in all treatments (Figure 8A, row 3), SE-Drug and SE-HIV promote the appearance of increased FA localization to membrane protrusions (Figure 8B, row 3). Vinculin is known to be activated downstream of cell surface receptors and binds talin and F-actin to provide a physical link between the actin cytoskeleton and integrins [94]. Thus, we examined the colocalization of F-actin with vinculin. Increased areas of F-actin•Vinculin were observed at AJs in all treatments (Figure 8A, row 4), with increased F-actin•Vinculin colocalization in AJs in cells treated with SE-Drug and SE-HIV (Figure 8C,D). Moreover, a preponderance of SE-Drug and SE-HIV treated cells contain F-actin•Vinculin in areas of cell protrusions (Figure 8B, row 4), but no significant difference was observed (Figure 8E,F). Although the role of SE in cell-to-matrix and cell-to-cell adhesions is yet to be determined, the increased detection of vinculin at areas of cell-to-matrix and cell-to-cell contacts suggest possible SE-mediated recruitment of vinculin to FAs and AJs in monocytes. These findings suggest that the SE-induced adhesive and protrusive modules may be functionally linked by the actin network to facilitate mechanotransduction as well as to provide cues between cells.

## 4. Discussion

In this study, we show that SEs are present in the semen of HIV-uninfected and HIV-infected participants independent of psychostimulants use. We also highlight the association of viral protein—RT and cocaine metabolite—benzoylecgonine with SE and show that SEs from study participants reprogrammed monocyte gene expression, morphometrics, and function.

Transcriptomics analysis of monocytes plated atop collagen and treated with SE from HIV−/+ and Drug−/+ groups showed that, in general, SE contain factors, such as COL16A1 and MMPs known to regulate cell adhesion [81,82,83] (Figure 2F and Figure 3C), cell chemotaxis (Figure 4A), and metalloprotease activity (Figure 4B). Furthermore, SE-Drug suppressed the expression of HUS1B, linked to the induction of cell death [85]. Amongst other observations was the result that SE-HIV suppressed the expression of TSC1, a protein known to maintain HIV in a latent state via the AKT-mTORC1 pathway [87]. These transcriptomic results support the observation that SE-Drug and SE-HIV promote monocyte adhesion, cytoskeletal reorganization, and chemotactic migration. 

Categorization of DEGs in each treatment into GO and KEGG functional terms identified a common theme—FA as a potential SE-regulated function (Table 8). FAs are large protein complexes that physically connect the ECM to the cytoskeleton of the cells and are integral to cell adhesion, signaling, actin cytoskeleton dynamics, and cell migration while SE induced monocyte adhesion to collagen, HIV infection, and/or psychostimulant use resulted in the secretion of SE-Drug and SE-HIV that potentiated monocyte adhesion to collagen (Figure 5). Indeed, HIV infection of lymphocytes and monocytes resulted in increased adhesion of the infected cells to vascular endothelium and ECM molecules [95], and treatment of monocytes with HIV Tat protein increased monocyte adhesion to endothelial monolayers [96].

Analyses of monocyte morphometrics and migration revealed that, in the presence of HIV infection and psychostimulant use, SE profoundly altered monocytes by increasing membrane ruffling and formation of filopodium-like structures. These changes resulted in variable modification of cell size and cell area by the different SEs; however, SE from HIV+Drug+ participants induced the most significant changes. These observations suggest that the ability of SE to regulate actin cytoskeleton dynamics, formation of membrane structures, FA, AJ, and localization of F-actin and vinculin to structures similar to the leading edge of migrating cells [97] and at cell–cell junctions depend on the microenvironment secreting the SE. On the basis of these observations, it is therefore possible that exosomes may modify FAs and AJs in response to changes in their microenvironment.

While FA bridges cells to ECM, AJ links neighboring cells and the actin–myosin cytoskeleton. These processes play a role in physiologic and pathologic signaling and cell migration and invasion during morphogenetics, tissue repair, and barrier disruption events [98,99]. In the center of these cell-to-cell and cell-to-ECM interactions is the actin machinery [100], which we found profoundly colocalized with vinculin in FA and AJ (Figure 8). Thus, elevated vinculin incorporation in the adhesion complexes may explain the observed enhanced firm adhesion of SE-Drug- and SE-HIV-treated monocytes to collagen and migration of monocytes to HIV secretome. 

The components of FA and AJ include scaffolding molecules, GTPases, and enzymes (kinases, phosphatases, proteases, and lipases). We found that, in comparison to vehicle-treated cells, monocytes treated with SE < SE-HIV < SE-Drug, in this order, released bioactive enzymes with enhanced gelatinolytic and caseinolytic activities. Amongst the enzymes identified by their molecular weight and banding pattern were gelatinolytic MMP2 and MMP9 and caseinolytic MMP7. However, other enzymes regulated by SE are yet to be identified. 

MMPs are vital to normal immune response to infection because they degrade the ECM for leukocyte migration and modulate the activity of cytokines, chemokines, and defensins. However, MMPs have been implicated in the upregulation of adhesion molecules [101] and in the immunopathology associated with tissue damage, metastasis, and microbial dissemination, such as bacterial meningitis, endotoxic shock, mycobacterial infection, and hepatitis B and HIV infection [102]. Our observation that SE-Drug and SE-HIV induced high levels of gelatinolytic and caseinolytic MMPs points to the potential that the use of psychostimulants and infection with HIV may change the function of SE. Indeed, it has been shown that HIV infection is associated with an altered production and secretion of MMPs which contribute to HIV-induced immunopathology, dysregulation of T-cell dynamics, leukocyte trafficking, and viral dissemination [72]. It is possible that the elevated MMP activity in HIV-infected cells may be related to HIV-associated immune activation, viral dissemination, and the development of HIV-associated diseases. In our studies, we observed that treatment of monocytes with SE from all clinical groups increased monocyte adhesion to collagen, with SE-Drug and SE-HIV producing the greatest increases (Figure 5). Indeed, HIV infection of lymphocytes and monocytes results in increased adhesion of the infected cells to vascular endothelium and ECM molecules [103] and treatment of monocytes with HIV Tat protein increased monocyte adhesion to endothelial monolayers [104]. It has been shown that MMPs, especially MMP9, promotes the ability of HIV-infected mononuclear cells to traverse artificial basement membrane barriers [105,106]. Similar to our observation of induction of MMP expression by SE, MMP9 is one of the immediate early genes expressed after HIV infection of monocyte/macrophage [107]. HIV proteins Tat and gp120 proteins upregulate MMP9 secretion from monocytes and T cells, while gp41-derived peptides stimulate MMP2 production [107,108]. Furthermore, elevated levels of MMP9 has been reported in vivo from patients with HIV infection not receiving ART [109]. Thus, factors in SE in general and in SE-Drug and SE-HIV, in particular, may play important roles in orchestrating monocytes dynamics in HIV infection and substances abuse.

Finally, because of the potential role of MMP in HIV dissemination via transmigration of infected cells [110] and HIV-associated pathologies (brain injury/neuronal damage, HIV-associated dementia (HAD) [111,112,113], Kaposi’s sarcoma (KS) [114,115,116], HIV-associated nephropathy (HIVAN) [117], and periodontal diseases [118,119]), understanding the role of SE-Drug and SE-HIV in the induction of MMP enzyme activity may constitute a novel therapeutic approach for HIV infection. 

Aside from the pathologic functions of monocytes, these cells perform systemic immune surveillance and maintenance of macrophage populations through constitutive migration from the bloodstream across the vascular endothelium. Thus, enhanced pathologic monocyte migration may promote disease while the consequence of losing constitutive migration may include defective cell-mediated immune responses. It remains to be determined whether alteration in monocyte morphometrics and motility induced by SE, SE-Drug, and SE-HIV will be pathologic or protective in primary monocytes. These interesting findings are limited by the possibility that HIV+Drug− and HIV+Drug+ SE may contain HIV particles because the semen samples were obtained before the advent of ART. Thus, HIV+ donors were almost certainly viremic. However, these limitations do not negate the findings of this study, since HIV particles and exosomes are naturally present in the semen of infected individuals. Hence, our findings highlight the possible events that may occur within individuals who are infected with HIV and use or do not use psychostimulants. Another caveat of this study is that the donors reported the use of multiple substances and alcohol. Thus, the observed effects may be due to one of the other substances or due to an interaction between different substances. Due to such complexities, well-controlled animal model studies are needed to determine drug-specific effects. Nevertheless, the presented data should pave the path for future works that study the effects of body fluids exosomes in the context of HIV infection and substance abuse. 

## Figures and Tables

**Table 1:** Description of psychostimulants and alcohol use by study participants.

Specimen ID	Hash/Marijuana Since Last Visit	Ever Used Hash/Marijuana	Poppers Since Last Visit	Ever Used Poppers	Crack/Cocaine Use Since Last Visit	Ever Used Crack or Cocaine	Uppers Since Last Visit	Ever Used Uppers	Ecstasy Since Last Visit	Ever used Ecstasy	Heroin/Opiates Since Last Visit	Ever Used Heroin/Opiates	PCP Since Last Visit	Downers Since Last Visit	Ethyl Chloride Since Last Visit	Unspecified Drug Since Last Visit	N. of drinks/wk Since Last Visit	Alcohol Use Since last Visit (drinks/wk)	Drinking Since Last Visit
**HIV−Drug−**																			
A1	N	N	N	N	N	N	N	N	N	N	N	N	N	N	N	N	0.866	1–3	Low/moderate
A2	N	N	N	N	N	N	N	N	N	N	N	N	N	N	N	N	0.347	1–3	Low/moderate
A3	N	N	N	N	N	N	N	N	N	N	N	N	N	N	N	N	0	0	None
A4	N	N	N	N	N	N	N	N	N	N	N	N	N	N	N	N	0	0	None
A5	N	N	N	N	N	N	N	N	N	N	N	N	N	N	N	N	2.25	1–3	Low/moderate
A6	N	N	N	N	N	N	N	N	N	N	N	N	N	N	N	N	0.245	1–3	Low/moderate
A7	N	N	N	N	N	N	N	N	N	N	N	N	N	N	N	N	0.245	1–3	Low/moderate
A8	N	N	N	N	N	N	N	N	N	N	N	N	N	N	N	N	2.25	1–3	Low/moderate
A9	N	N	N	N	N	N	N	N	N	N	N	N	N	N	N	N	5.25	4–13	Low/moderate
A10	N	N	N	N	N	N	N	N	N	N	N	N	N	N	N	N	0	0	None
A11	N	N	N	N	N	N	N	N	N	N	N	N	N	N	N	N	0	0	None
A12	N	N	N	N	N	N	N	N	N	N	N	N	N	N	N	N	5.25	4–13	Low/moderate
A13	N	N	N	N	N	N	N	N	N	N	N	N	N	N	N	N	0	0	None
A14	N	N	N	N	N	N	N	N	N	N	N	N	N	N	N	N	2.25	1–3	Low/moderate
A15	N	N	N	N	N	N	N	N	N	N	N	N	N	N	N	N	2.25	1–3	Low/moderate
A16	N	N	N	N	N	N	N	N	N	N	N	N	N	N	N	N	0.866	1–3	Low/moderate
**HIV−Drug+**																			
B1	Y	Y	Y	Y	Y	Y	N	N	N	N	N	N	N	N	N	N	24.5	>13	Moderate/heavy
B2	Y	Y	Y	Y	Y	Y	N	N	N	N	N	N	N	N	N	N	2.25	1–3	Low/moderate
B3	Y	Y	N	Y	Y	Y	N	N	N	N	N	N	N	N	N	N	10.5	4–13	Low/ moderate
B4	Y	Y	N	Y	Y	Y	N	N	N	N	N	N	N	N	N	N	12.25	4–13	Moderate/heavy
B5	Y	Y	N	Y	Y	Y	N	N	N	N	N	N	N	N	N	N	0.809	1–3	Low/moderate
B6	Y	Y	N	Y	Y	Y	Y	Y	N	N	N	N	N	N	N	N	12.25	4–13	Moderate/heavy
B7	Y	Y	N	Y	Y	Y	Y	Y	N	N	N	N	N	Y	N	N	5.25	4–13	Moderate/heavy
B8	Y	Y	N	Y	Y	Y	N	Y	N	N	Y	Y	N	Y	N	N	5.25	4–13	Moderate/heavy
B9	Y	Y	N	Y	Y	Y	N	Y	N	N	N	N	N	N	N	N	10.5	4–13	Low/moderate
B10	Y	Y	Y	Y	Y	Y	N	Y	N	N	N	N	N	Y	N	N	12.25	4–13	Moderate/heavy
B11	N	Y	N	Y	Y	Y	N	Y	N	N	N	N	N	N	N	N	2.25	1–3	Low/moderate
B12	Y	Y	N	Y	Y	Y	N	Y	N	Y	N	N	N	N	N	N	2.02	1–3	Moderate/heavy
B13	Y	Y	N	Y	Y	Y	N	N	N	N	N	Y	N	N	N	N	5.25	4–13	Low/moderate
B14	Y	Y	N	Y	Y	Y	N	N	N	N	N	Y	N	N	N	N	12	4–13	Binge
B15	Y	Y	Y	Y	Y	Y	Y	Y	Y	Y	N	N	N	Y	Y	N	56	>13	Binge
B16	Y	Y	N	Y	Y	Y	N	N	N	Y	N	N	N	N	N	N	0.866	1–3	Low/moderate
**HIV+Drug−**																			
C1	N	Y	N	Y	N	N	N	Y	N	N	N	N	N	N	N	N	5.25	4–13	Low/moderate
C2	N	Y	N	Y	N	N	N	N	N	N	N	N	N	N	N	N	5.25	4–13	Low/moderate
C3	N	Y	N	Y	N	Y	N	Y	N	Y	N	N	N	N	N	N	2.25	1–3	Low/moderate
C4	N	N	N	Y	N	N	N	N	N	N	N	N	N	N	N	N	0	0	None
C5	N	Y	N	N	N	N	N	N	N	N	N	N	N	N	N	N	0.866	1–3	Low/moderate
C6	N	Y	N	Y	N	N	N	N	N	N	N	N	N	N	N	N	2.25	1–3	Low/moderate
C7	N	Y	N	Y	N	N	N	N	N	N	N	N	N	N	N	N	5.25	4–13	Low/moderate
C8	N	Y	N	Y	N	N	N	N	N	N	N	N	N	N	N	N	0	0	None
C9	N	N	N	N	N	N	N	N	N	N	N	N	N	N	N	N	0	0	None
C10	N	Y	N	Y	N	Y	N	Y	N	N	N	N	N	N	N	N	0	0	None
C11	N	N	N	N	N	N	N	N	N	N	N	N	N	N	N	N	0	0	None
C12	N	N	N	N	N	N	N	N	N	N	N	N	N	N	N	N	0.866	1–3	Low/moderate
C13	N	N	N	N	N	N	N	N	N	N	N	N	N	N	N	N	5.25	4–13	Low/moderate
C14	N	Y	N	Y	N	N	N	N	N	N	N	N	N	N	N	N	0.347	1–3	Low/moderate
C15	N	Y	N	Y	N	Y	N	N	N	N	N	N	N	N	N	N	0	0	None
C16	N	Y	N	Y	N	N	N	N	N	N	N	N	N	N	N	N	0	0	None
**HIV+Drug+**																			
D1	Y	Y	Y	Y	Y	Y	Y	Y	Y	Y	N	N	N	N	Y	N	12.25	4–13	Moderate/heavy
D2	Y	Y	N	Y	Y	Y	N	Y	Y	Y	N	Y	N	N	N	Y	10.5	4–13	Low/moderate
D3	Y	Y	Y	Y	Y	Y	N	Y	N	Y	N	N	N	N	N	N	24.5	>13	Moderate/heavy
D4	Y	Y	Y	Y	Y	Y	N	N	N	N	N	N	N	N	N	N	24.5	>13	Moderate/heavy
D5	Y	Y	N	Y	Y	Y	N	Y	N	Y	N	N	N	N	N	N	24.5	>13	Moderate/heavy
D6	Y	Y	Y	Y	Y	Y	Y	Y	Y	Y	N	N	N	Y	Y	N	24.5	>13	Moderate/heavy
D7	Y	Y	N	Y	Y	Y	N	Y	N	N	N	N	N	N	N	N	2.25	1–3	Low/moderate
D8	Y	Y	Y	Y	Y	Y	N	Y	N	Y	N	N	N	N	N	N	2.25	1–3	Low/moderate
D9	Y	Y	Y	Y	Y	Y	N	N	N	N	N	N	N	N	N	N	5.25	4–13	Low/moderate
D10	N	Y	N	Y	Y	Y	N	Y	N	Y	N	N	Y	N	N	N	38.5	>13	Binge
D11	Y	Y	Y	Y	Y	Y	Y	Y	N	N	N	N	Y	Y	N	N	10.5	4–13	Low/moderate
D12	Y	Y	Y	Y	Y	Y	Y	Y	N	Y	N	N	N	N	Y	N	56	>13	Binge
D13	Y	Y	N	Y	Y	Y	N	Y	N	N	N	Y	Y	N	N	N	12.25	4–13	Moderate/heavy
D14	Y	Y	Y	Y	Y	Y	Y	Y	N	N	N	N	N	N	N	N	5.25	4–13	Moderate/heavy
D15	Y	Y	Y	Y	Y	Y	N	Y	N	Y	N	Y	N	N	N	N	12	4–13	Binge
D16	Y	Y	N	N	Y	Y	Y	Y	N	N	N	N	Y	N	N	N	2.02	1–3	Moderate/heavy

Y = Yes; N = No.

**Table 2:** Primer sequences.

Gene Symbol	Forward Primer	Reverse Primer
PTPRC	AATCTCCCTAGGCAGAGGCA	CCTCCCTCATGTGGCCAATT
ADGRG3	AGCGTATCACATGGCGAGAG	CCTGAGGGGAGGAGATTGGA
RELB	GATGGAGTCTCGCTCTGTCG	ATCCCAGCACTTTGAGAGGC
IL1B	ATGATGGCTTATTACAGTGGCAA	GTCGGAGATTCGTAGCTGGA
CD86	TATGGGCCGCACAAGTTTT	TCCTGTGGGCTTTTTGTGAT
MMP19	GAGGACTGGAGGCTGGAGTA	TGAAGGAGGGAGAGGGATGG
MMP9	TGAGGTGGTAGGATCGCTGA	ATGCCAGATCTCTGACCCCT
MMP1	AGTGGCCCAGTGGTTGAAAA	CCACATCAGGCAC
ELF1	TCCCAGCTATTCAGGAGGCT	CCCAAAGTTGCAGTGCAGTC
USP3	ATCCTCCCACCTCAGTCTCC	AGGCTGAGGTGGAGGATCAT
BBS4	GATGGAGTCTCGCTCTGTCG	AAATTAGCCGGGAGTGGTGG
TSC1	CTTGAGCTGGTGAGTGAGCA	GCGCTTGGCACTATTACTGC
EXOC4	CTGCCTCTGTACACGTGTGT	CGAGACAGCGAGACTCCATC
FGFR1	CAAAGGGGTGTGCGTTTCAG	TGGAGATGGGGTGGGAGTAG
TFAP4	CAGCGATTTCCGAATGCCTG	CAGCCTGGGCAACATAGTGA
ELK3	GTTTGTGACAGGCAGCACTG	CCTGGGGAGAGAAGGGATCA
FOSB	TTTTCTCCTCCGCCTGTGTC	TCACACTCTCACACTCGCAC
FOS	GCCCATTCCATCCCAACTCA	TGCCATCACCTCCATTCACC
FOXJ2	TAGAGGAGGGTGGGGTGATG	AGCCAGGCTCATAGTCAGGA
ERG	GACAACACAGCCAGCACTTG	CAGTTGTGCAAGTGTTCCCG
CREB1	TGCTGCACACATCATCCCAT	TAGATGGAGCTGGAGGCCTT
VTN	TCCCTGCCCATAGCTACAGT	AGGATCTCCCAGCATGAGGT
ATXN2L	GAGGGATGACTGGGAGGACT	CTAGTCCCTGCCCTAGGTGT
EGR1	CAGACCAGAAGCCCTTCCAG	TGGGTTTGATGAGCTGGGAC
YARS	CCTGTGTAAAGGCCCGGATT	CACAAACACGTGCTCACCAG
CD9	CCCACAAGGATGAGTTGATT	CAGCTTGTTGTAGGTGTCCTTG
POGZ	GTGCAGGACGTTGTCAACAC	GCCTCTCAAAGTGCTGGGAT

**Table 3:** Summary of the DEG in the four clinical groups (n = 3) as compared to vehicle ^1^.

	Genes Passed Filter Criteria ^1^	% of Total Genes ^2^	Upregulated		Downregulated	
			# of Genes	% of DEG	# of Genes	% of DEG
HIV−Drug−	293	1.37	177	60.41	116	39.59
HIV−Drug+	497	2.32	92	18.51	405	81.49
HIV+Drug−	641	2.99	179	27.93	462	72.07
HIV+Drug+	647	3.02	240	37.09	407	62.91

^1^ Filtration criteria FC < −2 or FC > 2 and *p*-value < 0.05 (Benjamini and Hochberg correction for multiple observations); ^2^ Total number of genes = 21448.

**Table 4:** Top 10 biological process GO terms from Webgestalt analysis ^1^.

	Gene Set	Description	# of Genes in Pathway	DEG in Pathway	Enrichment Score	*p*-Value
**SE-DEGs**	GO:0036230	granulocyte activation	500	8	8.775	2.01 × 10^−6^
	GO:0060326	cell chemotaxis	289	6	11.386	1.10 × 10^−5^
	GO:0002446	neutrophil mediated immunity	496	7	7.740	2.25 × 10^−5^
	GO:0097305	response to alcohol	231	5	11.870	5.40 × 10^−5^
	GO:0050900	leukocyte migration	419	6	7.853	8.85 × 10^−5^
	GO:0032103	positive regulation of response to external stimulus	293	5	9.358	1.66 × 10^−4^
	GO:0001525	angiogenesis	487	6	6.757	2.02 × 10^−4^
	GO:2000147	positive regulation of cell motility	493	6	6.674	2.15 × 10^−4^
	GO:0002521	leukocyte differentiation	496	6	6.634	2.23 × 10^−4^
	GO:0009636	response to toxic substance	499	6	6.594	2.30 × 10^−4^
**SE-Drug DEGs**	GO:1902115	regulation of organelle assembly	209	4	8.856	1.02 × 10^−3^
	GO:0140029	exocytic process	84	2	11.017	1.41 × 10^−2^
	GO:0043254	regulation of protein complex assembly	447	4	4.141	1.51 × 10^−2^
	GO:0071826	ribonucleoprotein complex subunit organization	245	3	5.666	1.56 × 10^−2^
	GO:0006289	nucleotide-excision repair	110	2	8.413	2.35 × 10^−2^
	GO:0006520	cellular amino acid metabolic process	318	3	4.365	3.07 × 10^−2^
	GO:0031023	microtubule organizing center organization	134	2	6.906	3.38 × 10^−2^
	GO:0071800	podosome assembly	20	1	23.136	4.24 × 10^−2^
	GO:0051383	kinetochore organization	21	1	22.034	4.44 × 10^−2^
**SE-HIV DEGs**	GO:0045576	mast cell activation	61	5	10.646	1.05 × 10^−4^
	GO:0071824	protein-DNA complex subunit organization	242	9	4.830	1.05 × 10^−4^
	GO:0002764	immune response-regulating signaling pathway	485	12	3.214	3.55 × 10^−4^
	GO:0002285	lymphocyte activation involved in immune response	172	7	5.286	3.66 × 10^−4^
	GO:0036230	granulocyte activation	500	12	3.117	4.67 × 10^−4^
	GO:0006959	humoral immune response	242	8	4.294	5.63 × 10^−4^
	GO:0002526	acute inflammatory response	154	6	5.060	1.22 × 10^−3^
	GO:0001525	angiogenesis	487	11	2.934	1.33 × 10^−3^
	GO:0002683	negative regulation of immune system process	416	10	3.122	1.39 × 10^−3^

^1^ Parameters for the enrichment analysis were as follows: Enrichment method: Over-representation Analysis (ORA), organism: hsapiens, enrichment categories: geneontology_Biological_Process_noRedundant, minimum number of IDs in the category: 5, maximum number of IDs in the category: 2000, False discovery rate (FDR) method: Benjamini and Hochberg, significance level: Top 10.

**Table 5:** Top 10 molecular function GO terms from Webgestalt analysis ^1^.

	Gene Set	Description	# of Genes in Pathway	DEG in Pathway	Enrichment Score	*p*-Value
**SE−DEGs**	GO:1990841	promoter-specific chromatin binding	46	2	26.787	2.48 × 10^−3^
	GO:0019199	transmembrane receptor protein kinase activity	81	2	15.212	7.51 × 10^−3^
	GO:0019838	growth factor binding	138	2	8.929	2.07 × 10^−2^
	GO:0001653	peptide receptor activity	149	2	8.270	2.39 × 10^−2^
	GO:0001228	DNA-binding transcription activator activity, RNA polymerase II-specific	444	3	4.163	3.36 × 10^−2^
	GO:0008237	metallopeptidase activity	185	2	6.661	3.57 × 10^−2^
	GO:1901567	fatty acid derivative binding	27	1	22.819	4.30 × 10^−2^
	GO:0017171	serine hydrolase activity	208	2	5.924	4.41 × 10^−2^
	GO:0043177	organic acid binding	212	2	5.812	4.57 × 10^−2^
	GO:0035035	histone acetyltransferase binding	29	1	21.245	4.61 × 10^−2^
**SE−Drug DEGs**	GO:0140098	catalytic activity, acting on RNA	350	3	3.912	4.02 × 10^−2^
	GO:0016701	oxidoreductase activity, acting on single donors with incorporation of molecular oxygen	28	1	16.299	5.96 × 10^−2^
	GO:0060090	molecular adaptor activity	194	2	4.705	6.69 × 10^−2^
	GO:0050839	cell adhesion molecule binding	478	3	2.864	8.53 × 10^−2^
	GO:1990841	promoter-specific chromatin binding	46	1	9.921	9.61 × 10^−2^
	GO:0097110	scaffold protein binding	58	1	7.868	1.20 × 10^−1^
	GO:0000049	tRNA binding	59	1	7.735	1.22 × 10^−1^
	GO:0070491	repressing transcription factor binding	73	1	6.252	1.48 × 10^−1^
	GO:0015631	tubulin binding	321	2	2.843	1.55 × 10^−1^
**SE−HIV DEGs**	GO:0019865	immunoglobulin binding	23	3	16.235	7.93 × 10^−4^
	GO:0001228	DNA−binding transcription activator activity, RNA polymerase II−specific	444	11	3.084	8.52 × 10^−4^
	GO:0016209	antioxidant activity	84	4	5.927	4.63 × 10^−3^
	GO:0070888	E-box binding	50	3	7.468	7.50 × 10^−3^
	GO:0016684	oxidoreductase activity, acting on peroxide as acceptor	55	3	6.789	9.76 × 10^−3^
	GO:0001227	DNA-binding transcription repressor activity, RNA polymerase II−specific	267	6	2.797	2.05 × 10^−2^
	GO:0060589	nucleoside-triphosphatase regulator activity	348	7	2.504	2.19 × 10^−2^
	GO:0005496	steroid binding	92	3	4.059	3.78 × 10^−2^
	GO:0015026	coreceptor activity	43	2	5.789	4.66 × 10^−2^

^1^ Parameters for the enrichment analysis were as follows: Enrichment method: ORA, organism: hsapiens, enrichment categories: geneontology_Molecular_Function_noRedundant, minimum number of IDs in the category: 5, maximum number of IDs in the category: 2000, FDR method: Benjamini and Hochberg, significance level: Top 10.

**Table 6:** Top 10 cellular component GO terms from Webgestalt analysis ^1^.

	Gene Set	Description	# of Genes in Pathway	DEG in Pathway	Enrichment Score	*p*-Value
**SE−DEGs**	GO:0030667	secretory granule membrane	293	5	9.554	1.26 × 10^−4^
	GO:0042581	specific granule	160	3	10.498	2.69 × 10^−3^
	GO:0070820	tertiary granule	163	3	10.305	2.84 × 10^−3^
	GO:0101002	ficolin-1-rich granule	183	3	9.179	3.93 × 10^−3^
	GO:0031012	extracellular matrix	496	4	4.515	1.02 × 10^−2^
	GO:0031904	endosome lumen	36	1	15.552	6.24 × 10^−2^
	GO:1903293	phosphatase complex	47	1	11.913	8.08 × 10^−2^
	GO:0005788	endoplasmic reticulum lumen	306	2	3.659	1.02 × 10^−1^
	GO:0001533	cornified envelope	65	1	8.614	1.10 × 10^−1^
	GO:0005766	primary lysosome	155	1	3.612	2.44 × 10^−1^
**SE−Drug DEGs**	GO:0030496	midbody	171	3	8.419	5.10 × 10^−3^
	GO:0005681	spliceosomal complex	176	3	8.180	5.52 × 10^−3^
	GO:0090734	site of DNA damage	66	2	14.543	8.19 × 10^−3^
	GO:0099023	tethering complex	67	2	14.326	8.43 × 10^−3^
	GO:0016607	nuclear speck	383	3	3.759	4.36 × 10^−2^
	GO:0044450	microtubule organizing center part	178	2	5.392	5.23 × 10^−2^
	GO:0035770	ribonucleoprotein granule	214	2	4.485	7.24 × 10^−2^
	GO:0005770	late endosome	242	2	3.966	8.95 × 10^−2^
	GO:0001917	photoreceptor inner segment	49	1	9.794	9.74 × 10^−2^
**SE−HIV DEGs**	GO:0030667	secretory granule membrane	293	8	3.127	3.98 × 10^−3^
	GO:0005766	primary lysosome	155	5	3.694	1.14 × 10^−2^
	GO:0042629	mast cell granule	22	2	10.411	1.55 × 10^−2^
	GO:0000790	nuclear chromatin	341	7	2.351	2.92 × 10^−2^
	GO:0016605	PML body	99	3	3.470	5.55 × 10^−2^
	GO:0005801	cis-Golgi network	60	2	3.817	9.64 × 10^−2^
	GO:0045177	apical part of cell	375	6	1.832	1.09 × 10^−1^
	GO:0034399	nuclear periphery	133	3	2.583	1.10 × 10^−1^
	GO:0042383	sarcolemma	134	3	2.564	1.12 × 10^−1^

^1^ Parameters for the enrichment analysis were as follows: Enrichment method: ORA, organism: hsapiens, enrichment categories: geneontology_Cellular_Component_noRedundant, minimum number of IDs in the category: 5, maximum number of IDs in the category: 2000, FDR method: Benjamini and Hochberg, significance level: Top 10.

**Table 7:** Top 10 KEGG pathways from Webgestalt analysis ^1^.

	Gene Set	Description	# of Genes in Pathway	DEG in Pathway	Enrichment Score	*p*-Value
**SE−DEGs**	hsa04657	IL-17 signaling pathway	93	4	17.374	6.69 × 10^−5^
	hsa05150	Staphylococcus aureus infection	56	3	21.640	3.25 × 10^−4^
	hsa05323	Rheumatoid arthritis	90	3	13.465	1.31 × 10^−3^
	hsa04928	Parathyroid hormone synthesis, secretion and action	106	3	11.432	2.10 × 10^−3^
	hsa04080	Neuroactive ligand-receptor interaction	277	4	5.833	4.13 × 10^−3^
	hsa05031	Amphetamine addiction	68	2	11.881	1.20 × 10^−2^
	hsa04917	Prolactin signaling pathway	70	2	11.541	1.27 × 10^−2^
	hsa04610	Complement and coagulation cascades	79	2	10.226	1.59 × 10^−2^
	hsa05132	Salmonella infection	86	2	9.394	1.87 × 10^−2^
	hsa05142	Chagas disease (American trypanosomiasis)	102	2	7.920	2.58 × 10^−2^
**SE−Drug DEGs**	hsa04914	Progesterone−mediated oocyte maturation	99	2	9.181	1.95 × 10^−2^
	hsa04931	Insulin resistance	107	2	8.494	2.25 × 10^−2^
	hsa04114	Oocyte meiosis	124	2	7.330	2.96 × 10^−2^
	hsa03040	Spliceosome	133	2	6.834	3.37 × 10^−2^
	hsa04910	Insulin signaling pathway	137	2	6.634	3.56 × 10^−2^
	hsa04150	mTOR signaling pathway	151	2	6.019	4.25 × 10^−2^
	hsa01523	Antifolate resistance	31	1	14.659	6.61 × 10^−2^
	hsa00260	Glycine, serine and threonine metabolism	40	1	11.361	8.46 × 10^−2^
	hsa00970	Aminoacyl-tRNA biosynthesis	44	1	10.328	9.26 × 10^−2^
**SE−HIV DEGs**	hsa05150	Staphylococcus aureus infection	56	5	9.836	1.41 × 10^−4^
	hsa04380	Osteoclast differentiation	128	5	4.303	5.93 × 10^−3^
	hsa05202	Transcriptional misregulation in cancer	186	6	3.554	6.58 × 10^−3^
	hsa05322	Systemic lupus erythematosus	133	5	4.142	6.95 × 10^−3^
	hsa00515	Mannose type O-glycan biosynthesis	23	2	9.580	1.82 × 10^−2^
	hsa04115	p53 signaling pathway	72	3	4.590	2.73 × 10^−2^
	hsa05166	Human T-cell leukemia virus 1 infection	255	6	2.592	2.75 × 10^−2^
	hsa00410	Beta-Alanine metabolism	31	2	7.108	3.19 × 10^−2^
	hsa04710	Circadian rhythm	31	2	7.108	3.19 × 10^−2^

^1^ Parameters for the enrichment analysis were as follows: Enrichment method: ORA, organism: hsapiens, enrichment categories: pathway_KEGG, minimum number of IDs in the category: 5, maximum number of IDs in the category: 2000, FDR method: Benjamini and Hochberg, significance level: Top 10.

**Table 8:** Top 10 KEGG pathways by count as determined by Transcriptome Analysis Console (TAC) software ^1^.

	Pathway	#Total	#Up	#Down	Significance	*p*-Value
**HIV-Drug-**	PI3K-Akt Signaling Pathway	20	13	7	0	1
	**Focal Adhesion**-PI3K-Akt-mTOR-signaling pathway	20	13	7	0.21	0.62234
	VEGFA-VEGFR2 Signaling Pathway	20	12	8	0.9	0.127165
	Nuclear Receptors Meta-Pathway	20	11	9	0.09	0.811647
	Regulation of toll-like receptor signaling pathway	19	10	9	2.84	0.001437
	MAPK Signaling Pathway	18	9	9	0.47	0.3394
	miR-targeted genes in muscle cell—TarBase	17	7	10	0.93	0.116256
	miR-targeted genes in lymphocytes—TarBase	17	7	10	2	0.009973
	Toll-like Receptor Signaling Pathway	15	9	6	2.96	0.001086
	Olfactory receptor activity	15	12	3	1	0.100786
**HIV-Drug+**	PI3K-Akt Signaling Pathway	35	8	27	0.11	0.783262
	miR-targeted genes in lymphocytes—TarBase	34	3	31	1.89	0.012864
	miR-targeted genes in muscle cell—TarBase	32	3	29	0.87	0.136266
	**Focal Adhesion**-PI3K-Akt-mTOR-signaling pathway	30	8	22	0.03	0.922794
	Olfactory receptor activity	24	8	16	1.82	0.015221
	Nuclear Receptors Meta-Pathway	24	6	18	0.8	0.157659
	VEGFA-VEGFR2 Signaling Pathway	22	7	15	0.08	0.827347
	miR-targeted genes in epithelium—TarBase	22	3	19	1.72	0.01896
	Genes related to primary cilium development (based on CRISPR)	21	1	20	2.73	0.001861
	Ciliary landscape	21	0	21	0	1
**HIV+Drug-**	miR-targeted genes in lymphocytes—TarBase	46	8	38	0.87	0.134124
	Nuclear Receptors Meta-Pathway	41	17	24	0.43	0.372732
	PI3K-Akt Signaling Pathway	40	14	26	0.14	0.728467
	miR-targeted genes in muscle cell—TarBase	38	8	30	0.74	0.183176
	MAPK Signaling Pathway	37	11	26	1.18	0.065447
	**Focal Adhesion**-PI3K-Akt-mTOR-signaling pathway	35	15	20	0.11	0.783054
	VEGFA-VEGFR2 Signaling Pathway	35	12	23	1.01	0.097823
	miR-targeted genes in epithelium—TarBase	29	7	22	1.06	0.087395
	Breast cancer pathway	26	8	18	1.54	0.029057
	Circadian rhythm related genes	26	7	19	0.3	0.50212
**HIV+Drug+**	Nuclear Receptors Meta-Pathway	43	29	14	0.67	0.211976
	PI3K-Akt Signaling Pathway	42	17	25	0.31	0.487261
	miR-targeted genes in lymphocytes—TarBase	42	9	33	1.42	0.038159
	**Focal Adhesion**-PI3K-Akt-mTOR-signaling pathway	37	16	21	0.28	0.520416
	VEGFA-VEGFR2 Signaling Pathway	36	18	18	1.2	0.062498
	miR-targeted genes in muscle cell—TarBase	34	10	24	1.38	0.041842
	miR-targeted genes in epithelium—TarBase	27	8	19	1.48	0.032898
	MAPK Signaling Pathway	26	11	15	0.08	0.838897
	TGF-beta Signaling Pathway	25	10	15	1.9	0.012623
	Ciliary landscape	23	8	15	0.04	0.913439

^1^ Gene expression parameters were as follows: Gene-level fold change < −2 or > 2; gene-level *p*-value < 0.05, gene-level FDR < 0.05, ANOVA statistical analysis with eBayes correction. The Pos/Neg Area Under the Curve (AUC) threshold was set to 0.7. Gene-level Signal Space Transformation-Robust Multi-Chip Analysis (SST-RMA) method was used for summarization.

## Supplementary Materials

The following are available online at https://www.mdpi.com/2073-4409/8/9/1027/s1, Figure S1: Bioanalyzer electropherograms of microarray samples for quality assessment.
