# Supplementary material for: Human Immunodeficiency Virus (HIV) Infection and Use of Illicit Substances Promote Secretion of Semen Exosomes that Enhance Monocyte Adhesion and Induce Actin Reorganization and Chemotactic Migration
Source: Cells. 2019 Sep 3;8(9):1027. doi: 10.3390/cells8091027 (PMC6770851; doi:10.3390/cells8091027)
Supplement: Supplementary file 1 [file cells-08-01027-s001.pdf]

Human immunodeficiency virus (HIV) infection and use of illicit substances promote secretion of semen exosomes that enhance monocyte adhesion, induce actin reorganization and chemotactic migration

Yuan Lyu[1], Hussein Kaddour[1], Steven Kopcho[1], Tyler D. Panzner[1], Nadia Shouman[1], Eun-Young Kim[2], Jeremy Martinson[3], Heather McKay[4], Otoniel Martinez-Maza[5], Joseph B. Margolick[6], Jack T. Stapleton[7], Chioma M. Okeoma[1]*

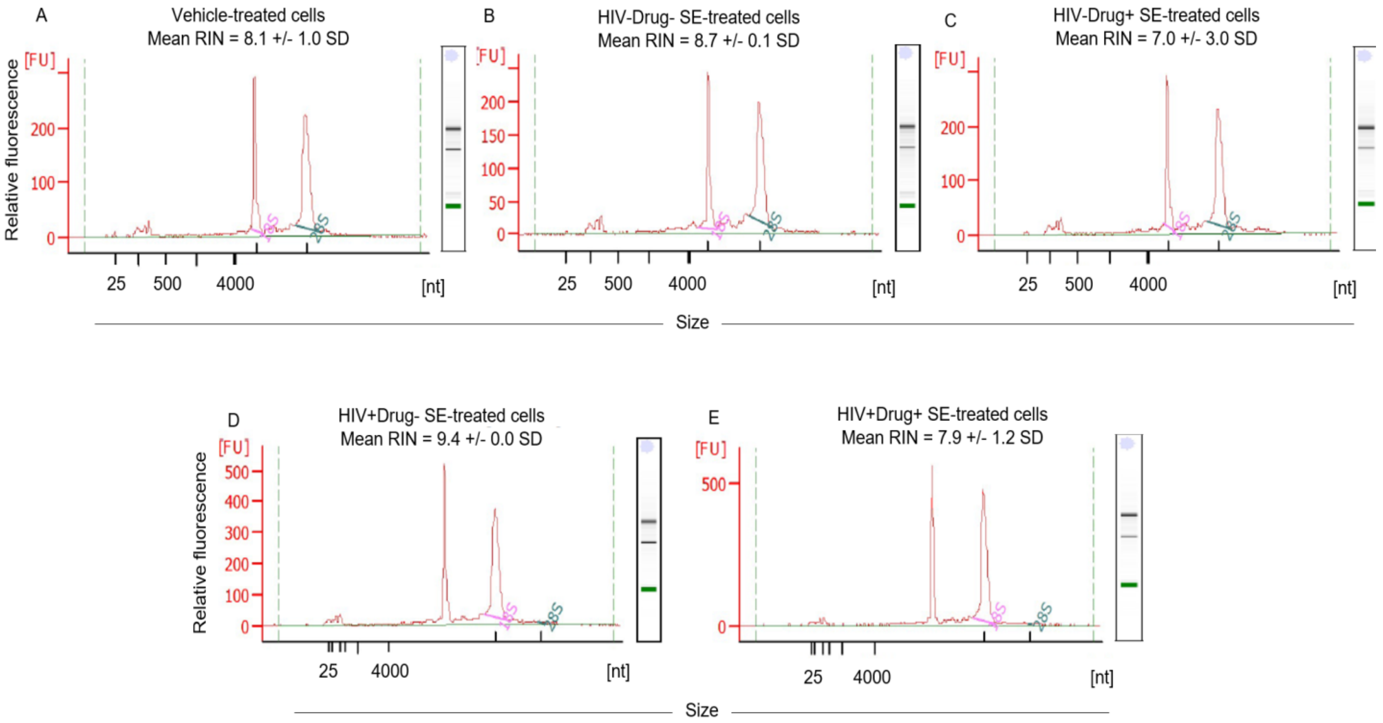

Supplemental Figure 1. Bioanalyzer electropherograms of microarray samples for quality assessment: (A-E) Representative electropherogram traces for 600 ng of RNA isolated from collagen-cultured monocytes that were treated with Vehicle (A), HIV-Drug- SE (B), HIV-Drug+ SE (C), HIV+Drug- SE (D), and HIV+Drug+ SE (E). The RNAs were applied to an RNA Nano Chip. RNA integrity and size were detected and analyzed on the Agilent 2100 Bioanalyzer with 2100 Bioanalyzer expert software (v B.02.08.S1648 (SR 1)). The "gel-like" images next to the electropherograms are digital images obtained from the Bioanalyzer extracted from conversion of electropherogram fluorescence traces using the Bioanalyzer software. Both electropherogram traces and "gel-like" images are the same sample from one representative experiment sample ran in parallel with other two samples (n=3 per group).
